# Hybridizing Feature Selection and Feature Learning Approaches in QSAR Modeling for Drug Discovery

**DOI:** 10.1038/s41598-017-02114-3

**Published:** 2017-05-25

**Authors:** Ignacio Ponzoni, Víctor Sebastián-Pérez, Carlos Requena-Triguero, Carlos Roca, María J. Martínez, Fiorella Cravero, Mónica F. Díaz, Juan A. Páez, Ramón Gómez Arrayás, Javier Adrio, Nuria E. Campillo

**Affiliations:** 1 Instituto de Ciencias e Ingeniería de la Computación (ICIC), Universidad Nacional del Sur-CONICET, San Andrés 800 – Campus Palihue, 8000 Bahía Blanca, Argentina; 2 Centro de Investigaciones Biológicas, Consejo Superior de Investigaciones Científicas (CSIC), Ramiro de Maeztu 9, 28040 Madrid, Spain; 3 Planta Piloto de Ingeniería Química (PLAPIQUI), Universidad Nacional del Sur-CONICET, Co. La Carrindanga km.7, CC 717 Bahía Blanca, Argentina; 4 Instituto de Química Médica, Consejo Superior de Investigaciones Científicas (CSIC), Juan de la Cierva 3, 28006 Madrid, Spain; 5 Departamento de Química Orgánica, Universidad Autónoma de Madrid (UAM). Cantoblanco, 28049 Madrid, Spain; 6 Institute for Advanced Research in Chemical Sciences (IAdChem), UAM, 28049 Madrid, Spain

## Abstract

Quantitative structure–activity relationship modeling using machine learning techniques constitutes a complex computational problem, where the identification of the most informative molecular descriptors for predicting a specific target property plays a critical role. Two main general approaches can be used for this modeling procedure: feature selection and feature learning. In this paper, a performance comparative study of two state-of-art methods related to these two approaches is carried out. In particular, regression and classification models for three different issues are inferred using both methods under different experimental scenarios: two drug-like properties, such as blood-brain-barrier and human intestinal absorption, and enantiomeric excess, as a measurement of purity used for chiral substances. Beyond the contrastive analysis of feature selection and feature learning methods as competitive approaches, the hybridization of these strategies is also evaluated based on previous results obtained in material sciences. From the experimental results, it can be concluded that there is not a clear winner between both approaches because the performance depends on the characteristics of the compound databases used for modeling. Nevertheless, in several cases, it was observed that the accuracy of the models can be improved by combining both approaches when the molecular descriptor sets provided by feature selection and feature learning contain complementary information.

## Introduction

Quantitative structure–activity/property relationship (QSAR/QSPR) models characterize the associations among molecular descriptors that represent information related to the structure of chemical compounds and a target physicochemical or biological property under study. These models play a central role in drug identification or optimization of drugs because they allow a preliminary *in silico* evaluation of crucial properties related to the activity, selectivity, and toxicity of candidate molecules^1–3^. In this way, important savings in terms of money and time can be achieving during the drug discovery projects and therefore be more efficient^4^.

QSAR models can be defined as regression or classification models by means of using different computational strategies, as statistical methods or artificial intelligence approaches among others. In particular, machine learning methods (such as artificial neural networks) had become extensively used in this field during the last decades^5–7^. Several computational issues must be addressed when QSAR models are inferred by machine learning methods. One of these problems is deciding which molecular descriptors should be used for defining a particular QSAR model. This decision depends on the structural information captured per each molecular descriptor and the characteristics of the target property. In this context, there are two main approaches for dealing with this task: feature selection and feature learning methods.

Feature selection strategies obtain a reduced set of molecular descriptors from a high quantity of them, previously calculated by using computational tools (like Dragon^8^ or Padel^9^). In other words, these methods solve a combinatorial optimization problem, where alternative subsets of molecular descriptors are selected and evaluated in order to identify a group of descriptors well-correlated with a target property. Several studies have demonstrated the benefits of using feature selection in drug design^10^. Nevertheless, most of these approaches require high computational effort for evaluating alternative combinations of molecular descriptors. For example, Dragon allows to compute thousands of different descriptors, but the number of variables needed for obtaining an accurate QSAR model is usually very low. Therefore, the number of combinations of descriptor subsets to be explored by feature selection methods is commonly huge.

In contrast, feature learning methods avoid the use of a combinatorial exploration procedure. These approaches extract a reduced number of new features directly calculated from the chemical structure of the compounds, without using traditional molecular descriptors calculated by software tools. After the extraction, QSAR models can be directly inferred from these learned features. In this way, no procedure for selecting descriptors is required by these strategies. Nevertheless, even when these methods run with low computing times, they are mostly constrained to the extraction of 2D molecular features of the compounds. Another important issue is related to the chemical interpretability of QSAR models because extracted variables obtained by some feature learning strategies (like principal component analysis) are hard to understand in molecular terms.

Taking into account the advantages and limitations of feature selection and feature learning approaches, an experimental study for contrasting both strategies in the inference of regression and classification QSAR models may result enlightening for the virtual screening practitioners. This constitutes the primary goal of this work: to evaluate both methodologies under different experimental scenarios using two state-or-art software tools (DELPHOS^11^ and CODES-TSAR^12^) as paradigms of these main approaches. DELPHOS is based on the feature selection method for QSAR modeling developed by Soto *et al*.^13^. The method splits the feature selection task into two sequential phases as a strategy for maintaining a reasonable computational effort without losing accuracy on the final QSAR models. DELPHOS has successfully been used in QSAR modeling applied to different application domains, such as virtual screening of drugs^13^, environmental sciences^14^, and material sciences^15, 16^. On the other hand, CODES-TSAR is a state-of-art feature learning method specifically designed for QSAR modeling. It creates a numerical definition capable of capturing the whole molecule representation. The descriptors generated by CODES do not refer to any specific feature; then they can be used to perform the prediction of any desirable property. The method is based on neural computing, and it enables the easy generation of the required numerical descriptors of the structures involved in the study with the only knowledge of their SMILES code (i.e., their chemical structure) by applying a dimensionality reduction method based on artificial neural networks. CODES does not need 3D information since the topological space and its later conversion to a neural space only need details about points and their relationships; which is the chemical structure in itself. CODES-TSAR manages to identify non-linear relationships in the regression models and it is successfully used for inferring QSAR models in different drug design problems^17–19^.

However, these methods should not only be considered as alternative approaches (mutually excluding strategies); instead, it is also interesting to assess potential benefits obtained from a combination of them. In this respect, there is a recent antecedent where QSAR models generated from molecular descriptors suggested by both methods achieved a higher precision than QSAR models inferred by DELPHOS and CODES-TSAR alone^16^. In that work, QSAR models for predicting a mechanical property of polymeric materials were reported. It was observed that the sets of descriptors obtained by both techniques provide complementary and relevant information for the inference of the target property. Therefore, a secondary aim of this work is to assess if the descriptors provided by these competing methodologies have a complementary nature that can help to achieve more accurate QSAR models in the context of virtual screening of drugs.

Keeping the above in mind, we propose to study the combination of both approaches to generate QSAR models for three different physicochemical issues: blood-brain-barrier (BBB), oral absorption determined as human intestinal absorption (HIA) and enantiomeric excess (EE). In each case, several machine learning approaches were tested for inferring QSAR models (regression and classification models) from the molecular descriptors obtained by DELPHOS and CODES-TSAR. The most accurate QSAR models obtained from these experiments have been analyzed, from a mathematical and chemical perspective, in order to contrast the strengths and weaknesses of both approaches.

## Results

**Figure 1:**
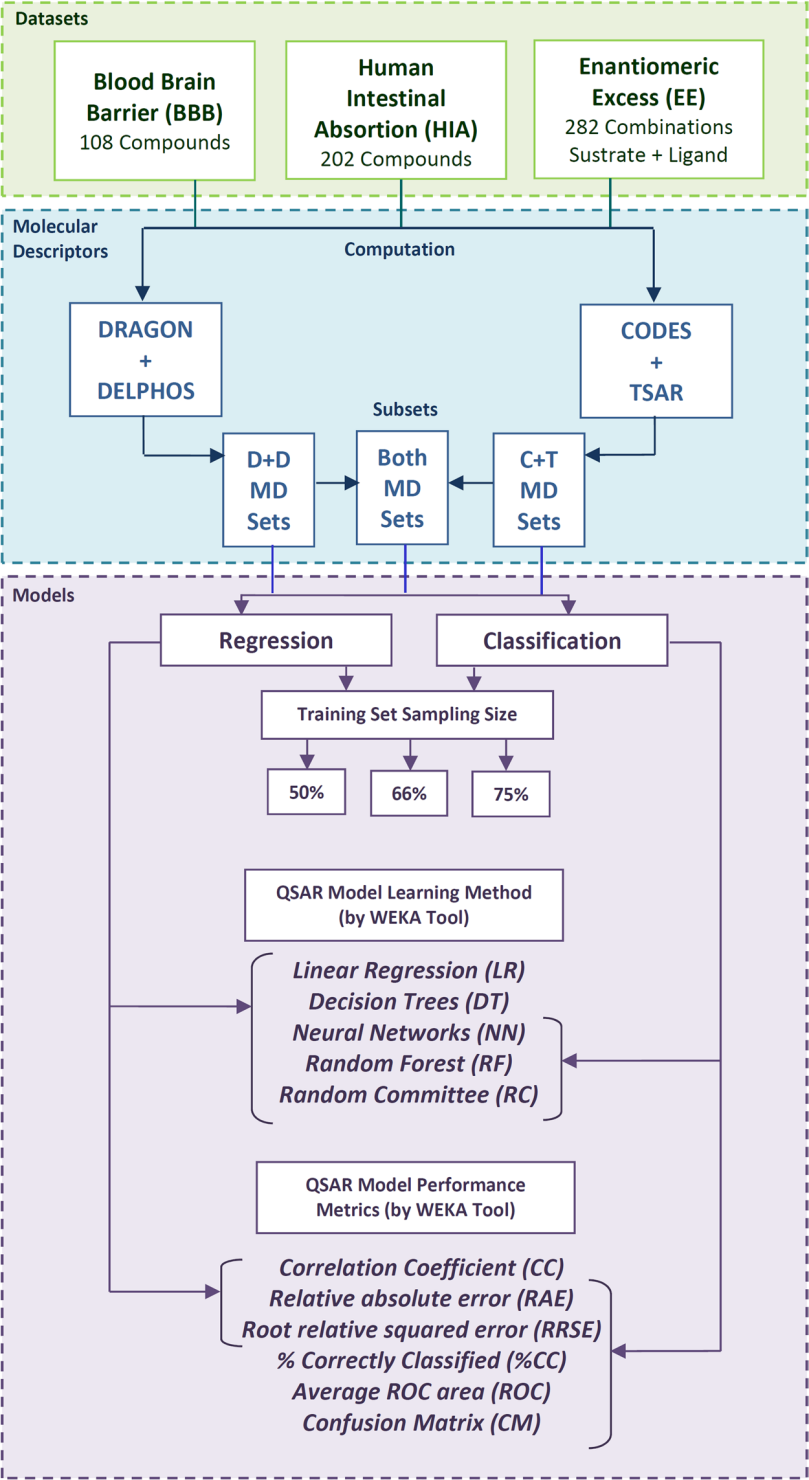
Scheme of the *in silico* experiments reported for the prediction of blood-brain-barrier (BBB), human intestinal absorption (HIA) and enantiomeric excess (EE).

In this section, several QSAR models inferred by feature selection and feature learning for different physicochemical properties are described. Figure 1 presents a scheme of the experiments design. Three databases are used in the experiments: blood-brain-barrier (BBB), human intestinal absorption (HIA) and enantiomeric excess (EE). For each dataset, molecular descriptors are computed following two different approaches: feature selection and feature learning. In the first case, DRAGON software was executed over the dataset, excluding the calculation of 3D molecular descriptors. We decided to compute only 0D, 1D, and 2D descriptors in order to achieve a fair comparison between both approaches since CODES-TSAR does not capture 3D molecular features. From the set of descriptors returned by DRAGON, feature selection step is executed by DELPHOS tool. In the second case, CODES tool processes each molecule structure contained in the dataset codifying a dynamic matrix. This matrix is the input used by the TSAR software, which finally computes the molecular descriptors (MD) for each compound. In this way, different subsets of descriptors are obtained using DRAGON with DELPHOS (*D* − *D MD Sets*) and CODES with TSAR (*C* − *T MD Sets*). Combined subsets, which integrate the features computed by both methods, are also defined (*Both MD Sets*). After that, regression and classification QSAR models are inferred from these molecular descriptor subsets.

The discretization thresholds of target property values used for defining classes (for classification models) are detailed in the Material and Methods section. The models are computed by WEKA^20^ using alternative inference methods: Linear Regression (LR), Decision Trees (DT), Neural Networks (NN), Random Forest (RF) and Random Committee (RC). We decided to test with several methods for inferring the QSAR models because recent studies have shown that there does not exist a more advisable strategy for inferring the QSAR from the subsets of descriptors^21^. For each inference method, the parameter settings provided by default for WEKA were used in the experiments. Regarding the performance assessment, several metrics were computed using WEKA. For regression models, the correlation coefficient (CC), relative absolute error (RAE) and root relative square error (RRSE) are reported. For classification models, the percentage of cases correctly classified (%CC), average Receiver Operating Characteristic (ROC) area and confusion matrix (CM), together with the relative absolute error (RAE) and root relative square error (RRSE), are informed. Finally, different QSAR models were inferred varying the splitting rates used for defining the training and testing set sizes (50/50, 66/34, 75/25). In all cases, the stratified sampling provided per default by WEKA was used.

In Table 1, a summary of the best QSAR models obtained in these experiments is reported. For each dataset, performance metrics (CC, %CC, ROC), training conditions (sampling sizes and QSAR learning method) and the strategy used for obtaining the molecular descriptor subset (D + D, C − T, Both) are indicated for the best regression and classification model inferred during the experiments. A detailed analysis of these QSAR models is presented in the next Subsections.

**Table 1:** Metrics of the best QSAR models for each dataset.

Dataset	Best Regression QSAR Model				Best Classification QSAR Model				
	CC	% Training Sampling Size	Mol. D. Subset	Learning Method	%CC	ROC	% Training Sampling Size	Mol. D. Subset	Learning Method
BBB	0.76	66%	D + D	R. Committee	86.49%	0.720	66%	D + D	N. Networks
HIA	0.75	75%	Both	N. Networks	86.96%	0.865	66%	D + D	R. Forest
EE	0.69	75%	C − T	R. Forest	81.43%	0.678	75%	C − T	R. Forest

### QSAR models for blood-brain-barrier (BBB)

#### Data set

Regarding prediction of blood-brain barrier permeation, we have used a dataset with 108 compounds with known logBB values, previously published by our group^17^.

#### Drug-like properties calculation and similarity assessment

An important step in QSAR/QSPR studies is to have a structural diversity set of compounds in order to have a representative structural diversity space. In addition, we have wanted to have the datasets characterize from a drug-like point of view. Thus, we use two different approaches to analyse the diversity of our datasets, from a structural and drug-like point of view.

Considering that CODES encodes a structure from the chemical structure of the molecule based on the atom nature, the number of atom bonds and the connectivity with the rest of the molecule, we have used these descriptors as measure of structural diversity. The similarity analysis for every dataset was performed using Pearson Similarity index. The correlations were transformed and rescaled to a 0–1 range obtaining diverse datasets. The similarity indexes for the BBB dataset (Supplementary Table S1) were ordered increasingly from 0 to 1. As it can be observed, the compounds in the dataset present a wide range of structural diversity.

**Figure 2:**
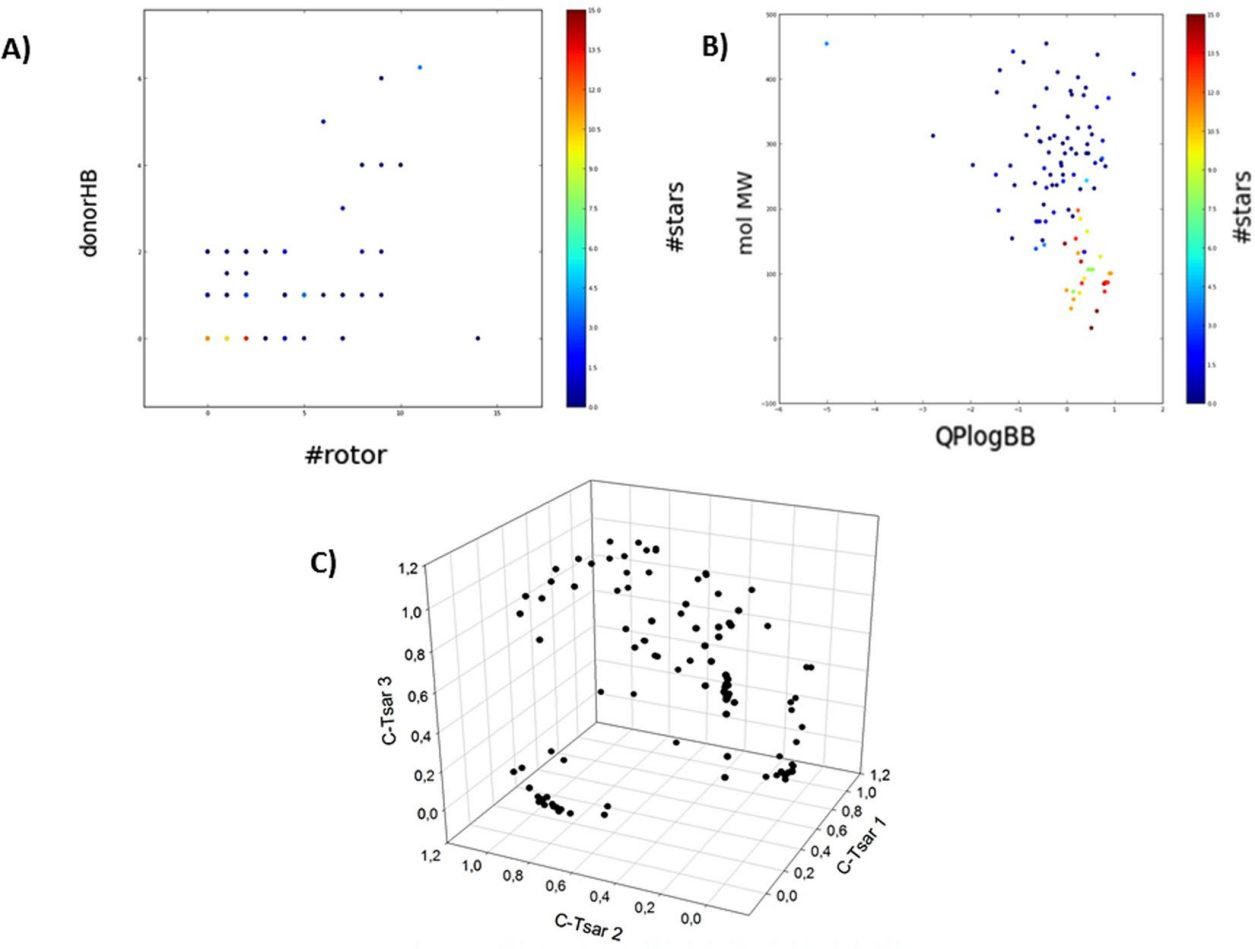
Physicochemical and structural representation of the BBB dataset. (**A**) Dispersion of compounds regarding rotatable bonds and hydrogen bond donors. Colors are defined by stars, a parameter related to physical-chemical properties of commercially available drugs. (**B**) Dispersion of the dataset taking into account molecular weight theoretical value of blood barrier permeability. The color is defined by the parameter stars. (**C**) Structural diversity represented as a 3D dispersion of compounds regarding CODES descriptors.

Regarding the drug-like characterization, the data set was treated with Qikprop^22^, calculating their physicochemical and drug-like properties. The most representative descriptors obtained have been analyzed and plotted to show the drug-like characterization (Fig. 2). Here, we found that molecular weight values have a range between 16 and 455 Da, while logBBB values go from −2.51 to 5.85. These wide ranges of values include compounds that are totally able to cross the BBB and compounds unable to enter in the CNS. Hydrogen bond donors and acceptors have also a wide range of values; the former is between 0 and 14, while the latter presents a range between 0 and 11. Molecules in this dataset have from 0 rotatable bonds to 14.

An additional important feature to analyze in a dataset is the number of compounds that are within the limits of significant drug-like properties. Thus, the Lipinski’s criteria, generally referred to as the “rule of 5”, has been used for the evaluation of these properties, finding that only a 7.41% of the compounds violate one or more Lipinski’s rules^23^. Furthermore, the stars parameter is a Qikprop index directly related with drug-like properties and indicates the number of property or descriptor values that fall outside the 95% range of similar values for known drugs. As we can observe in Fig. 2, most of the compounds included in the dataset present a star index below 5, which constitutes a value recommended for drug-like compounds. Therefore, it is possible to affirm that the BBB dataset is a diverse collection of compounds from a physicochemical and drug-like property point of view that can be further used in the predictive models.

#### Molecular Descriptor Subsets selection and QSAR models inference

The experiments were designed following the procedure described in Fig. 1. It is important to mention that DELPHOS infers multiple alternative selections of molecular descriptors for defining a QSAR model. In this case, twenty-five putative subsets have been computed. From them, we decided to pick the two subsets with the lowest RAE values reported by DELPHOS, which correspond to M2_BBB_ and M13_BBB_ subsets. Therefore, for the BBB dataset, five alternative subsets of molecular descriptors were used for the experiments: one subset obtained from CODES-TSAR (CT_BBB_), two subsets selected by DRAGON/DELPHOS (M2_BBB_ and M13_BBB_) and the union of M2_BBB_ and M13_BBB_ subsets with CT_BBB_ (M2_BBB_ ∪ CT_BBB_ and M13_BBB_ ∪ CT_BBB_). Table 2 summarizes the characteristics of these subsets.

**Table 2:** Molecular descriptors subsets used for inferring the blood-brain-barrier QSAR models.

Subset Name	Method	Size	Molecular descriptor names
CT_BBB_	CODES-TSAR	3	CODES-T1, CODES-T2, CODES-T3
M2_BBB_	D/DELPHOS	3	nR06, SIC1, CIC5
M13_BBB_	D/DELPHOS	7	AMW, RBN, MATS5e, MATS4p, EEig12d, JGI7, Hy
M2_BBB_ ∪ CT_BBB_	Combined	6	nR06, SIC1, CIC5, CODES-T1, CODES-T2, CODES-T3
M13_BBB_ ∪ CT_BBB_	Combined	10	AMW, RBN, MATS5e, MATS4p, EEig12d, JGI7, Hy, CODES-T1, CODES-T2, CODES-T3

Using these descriptor subsets, several regression and classification QSAR models were inferred applying different machine learning approaches. The accuracy metric values achieved per each QSAR model are reported in Supplementary Table S2 and all CSV files used for running the BBB experiments in WEKA are made available as Supplementary Zipped File 1. The regression and classification QSAR models with better performance were obtained using the M13_BBB_ subset selected by DELPHOS. In particular, the best classification model obtained from the M13_BBB_ subset achieves an accuracy of 86.49%. For the BBB classification experiments, three classes were defined: molecules which cross the BBB, molecules which do not cross the BBB and a gray area that represents uncertainty (see discretization thresholds in Materials and Methods section). From the confusion matrix, we can observe that this QSAR model has a high precision for compounds which cross the blood-brain-barrier (85.71%) and compounds which do not cross the blood-brain-barrier (100%). Nonetheless, the classifier accuracy decreases for compounds in the gray zone. The performance falloff for this intermediate class can be related to the class imbalance in the testing set, because only 10.81% of samples corresponds to molecules in the gray zone. This fact can also explain the moderate value of the average ROC area (0.72).

Some of the descriptors found in M13_BBB_ are related to the constitutional indices, such as average molecular weight (AMW) or a number of rotatable bonds (RBN). These constitutional indices, also known as 0D-descriptors, are obtained from the chemical formula, as they do not consider the tridimensional structure of the ligands. Another important family of descriptors found was the 2D autocorrelation. Moreover, two out of three descriptors in this family are in relation with the Moran coefficient (MATS4P, MATS5e) regarding polarizability and Sanderson electronegativity, respectively. The other descriptor of this family involved in the model is JGI7, and it is related to the topological charge. Finally, two more descriptors are present in the model, EEig12 d and Hy, which are part of edge adjacency indices and molecular properties families, respectively. The former is in relation with dipole moments, while the latter has a direct relation with the hydrophobicity of the molecules. In summary, in physicochemical terms, some of these descriptors are in direct relation with well-known properties of molecules that allow or prevent the compounds to pass the blood brain barrier, such as molecular weight or logP. It has been extensively probed that compounds with higher logP values are more likely to pass the BBB, while compounds with low logP values have difficulties to cross the barrier. In a similar way, compounds that show very high molecular weight usually are not likely to cross the blood brain barrier. Also, parameters like polarizability, Sanderson electronegativity, dipole moments and topological charge are in relation with hydrophobicity and charge distribution of the molecules, thus affecting their capacity to cross the BBB.

**Figure 3:**
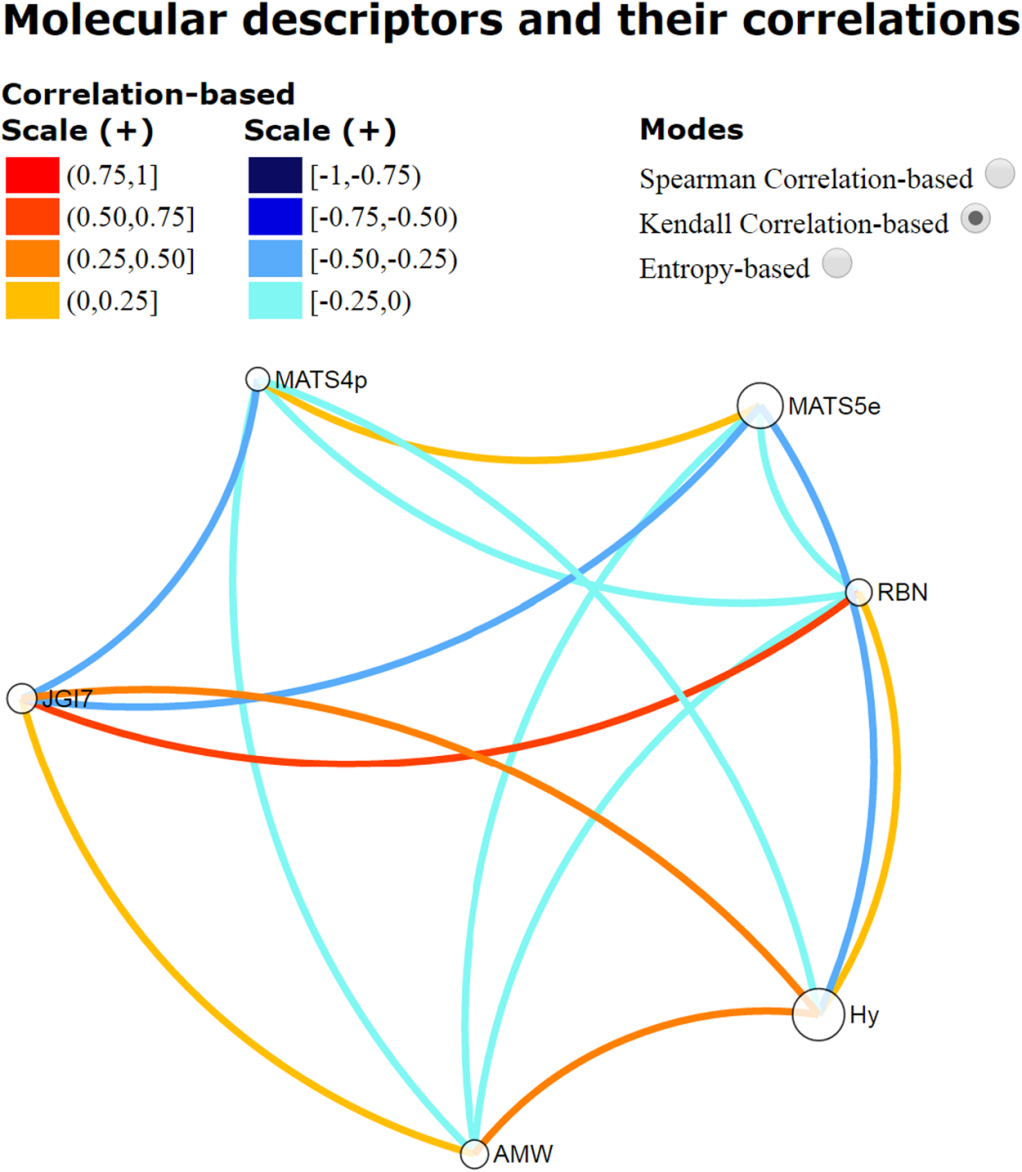
Redundancy analysis among the molecular descriptors that conforms the model M13_BBB_.

Likewise, we analyzed the relationship among the descriptors in statistical terms by using VIDEAN^24^, which is a visual analytics tool for the study of molecular descriptor subsets. The analysis of the correlation between the seven descriptors that conform the model M13_BBB_ is presented in Fig. 3. Spearman Correlation-based was used, but it possible to choose other two correlation modes. In all cases, the goal was to identify models with low correlation among descriptors that means low redundant information. This can be observed by light tones of different colors depending on the mode. Figure 3 shows the relationships (edges) among the seven descriptors (nodes), and there can be seen links with light tones of orange and blue, demonstrating the low data redundancy in this model (see both scales (+) and (−)).

In summary, we can assert that for the BBB dataset the best QSAR models obtained by DELPHOS achieve a reasonable prediction accuracy. In this regard, the molecular descriptors chosen by the feature selection method are clearly related with the logBBB in physicochemical terms and they contain low redundancy levels. From the complete set of experiments reported on the Supplementary Table S2, we computed confidence intervals in order to determine if there are differences with statistical significance in the performance of both approaches. The results reveal that DRAGON/DELPHOS approach outperforms CODES-TSAR with statistical significance in both QSAR modeling strategies, regression and classification, for this dataset.

### QSAR models for human intestinal absorption (HIA)

#### Dataset

Regarding prediction of human intestinal absorption, we have used a dataset with 202 compounds with known HIA values previously published by our group^19^.

#### Drug-like properties calculation and similarity assessment

**Figure 4:**
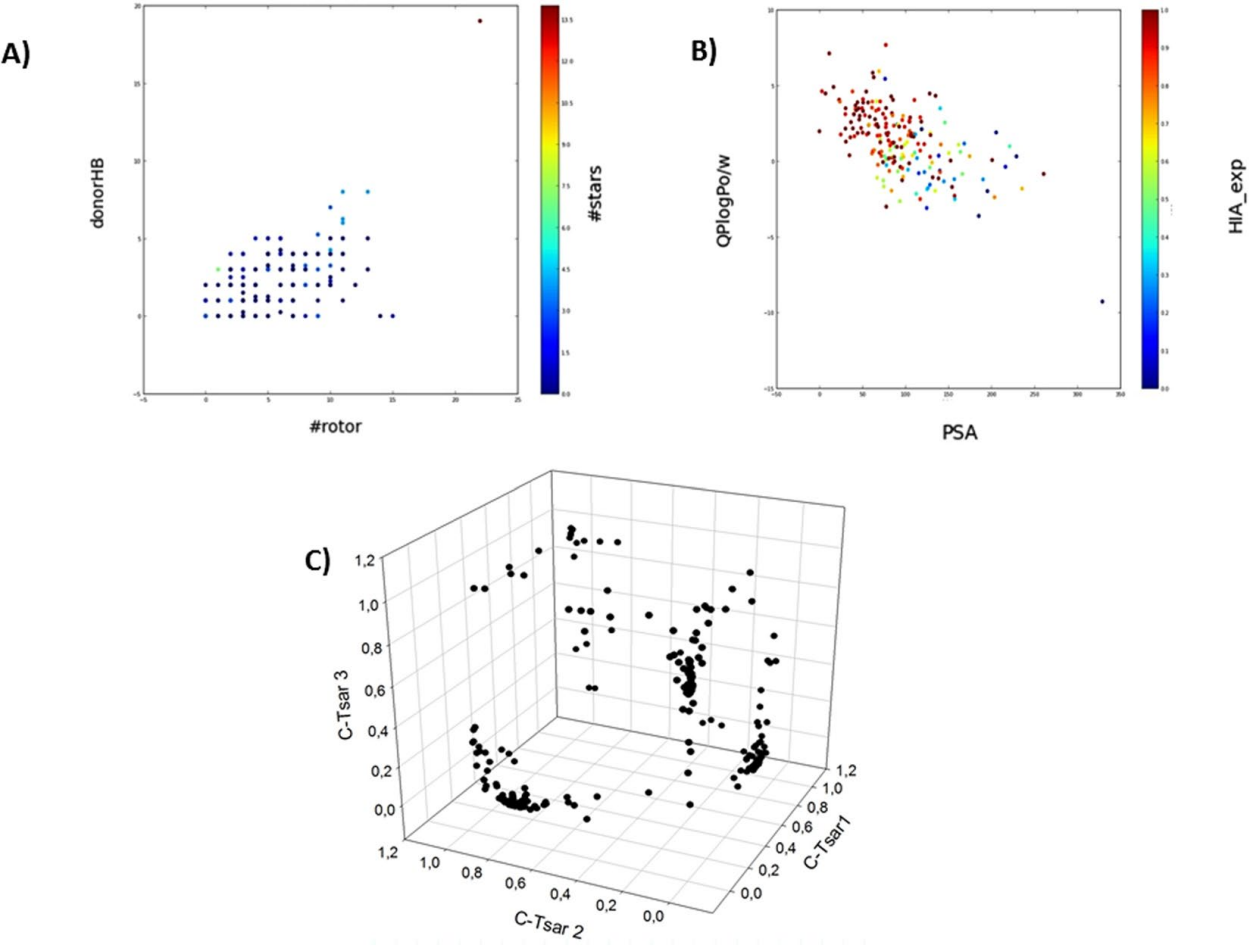
Physicochemical and structural representation of the HIA dataset. (**A**) Dispersion of the compounds regarding hydrogen bond donors and rotatable bonds. Color is defined by stars, a parameter related to physical-chemical properties of commercially available drugs. (**B**) Dispersion of the dataset taking into account logP values and Polar Surface Area (PSA). Color is defined by HIA experimental values. (**C**) Structural diversity represented as a 3D dispersion of compounds using CODES descriptors.

The similarity indices for HIA dataset using CODES-TSAR descriptors are shown in Supplementary Table S3. Therefore, in a similar way of that of the previous dataset, it can be observed that the compounds in the dataset present a wide range of diversity. Physicochemical properties of this dataset were calculated using Qikprop and the most representative descriptors were analyzed in order to show the diversity of the dataset. All this information is depicted in Fig. 4.

Molecular weight (MW), polar surface area (PSA), hydrogen bond donors (HBD), hydrogen bond acceptors (HBA) or the number of violations for the Lipinski rules are some of the descriptors that were plotted to show the different physicochemical properties. Molecular weight and logP values show a wide distribution, essential for a diverse database to be further used. The molecular weight range goes from 75 to 614 Da, and the logP distribution is even broader, with a minimum of −9.27 and a maximum of 7.67. The range of hydrogen bonds donors is between 0 and 19, hydrogen bonds acceptors go from 0 to 28 and the number of rotatable bonds varies from 0 to 15. Regarding the Lipinski’s rule, most of the compounds fulfill the Lipinski criteria and only a 10.89% of them violates one or more Lipinski’s rules. This fact confirms a diverse dataset, useful to train our model for obtaining the best possible prediction for a wide diversity of compounds, and to be further applied to drug-like molecules.

#### Molecular Descriptor Subsets selection and QSAR models inference

Regarding the subsets of molecular descriptors obtained for HIA dataset, we decided to take the two subsets with lower RAE values reported by DELPHOS (the same criteria applied before for BBB dataset), which correspond to M5_HIA_ and M9_HIA_ subsets. Therefore, for HIA dataset, five alternative subsets of molecular descriptors were used for the experiments: one subset obtained from CODES-TSAR (CT_HIA_), two subsets selected by DRAGON-DELPHOS (M5_HIA_ and M9_HIA_) and the union of M5_HIA_ and M9_HIA_ subsets with CT_HIA_ (M5_HIA_ ∪ CT_HIA_ and M9_HIA_ ∪ CT_HIA_). Table 3 summarizes the characteristics of these subsets.

**Table 3:** Molecular descriptor subsets used for inferring the human intestinal absorption QSAR models.

Subset Name	Method	Size	Molecular descriptor names
CT_HIA_	CODES-TSAR	3	CODES-T1, CODES-T2, CODES-T3
M5_HIA_	D/DELPHOS	4	AMW, MATS7m, ESpm01d, TPSA(NO)
M9_HIA_	D/DELPHOS	5	AMW, GATS6v, JGI4, VRp2, TPSA(NO)
M5_HIA_ ∪ CT_HIA_	Combined	7	AMW, MATS7m, ESpm01d, TPSA(NO), CODES-T1, CODES-T2, CODES-T3
M9_HIA_ ∪ CT_HIA_	Combined	8	AMW, GATS6v, JGI4, VRp2, TPSA(NO), CODES-T1, CODES-T2, CODES-T3

Using these descriptor subsets, several regression and classification QSAR models were inferred applying different machine learning approaches. The accuracy metric values achieved per each QSAR model are reported in Supplementary Table S4 and all CSV files used for running the HIA experiments in WEKA are available as Supplementary Zipped File 2.

**Figure 5:**
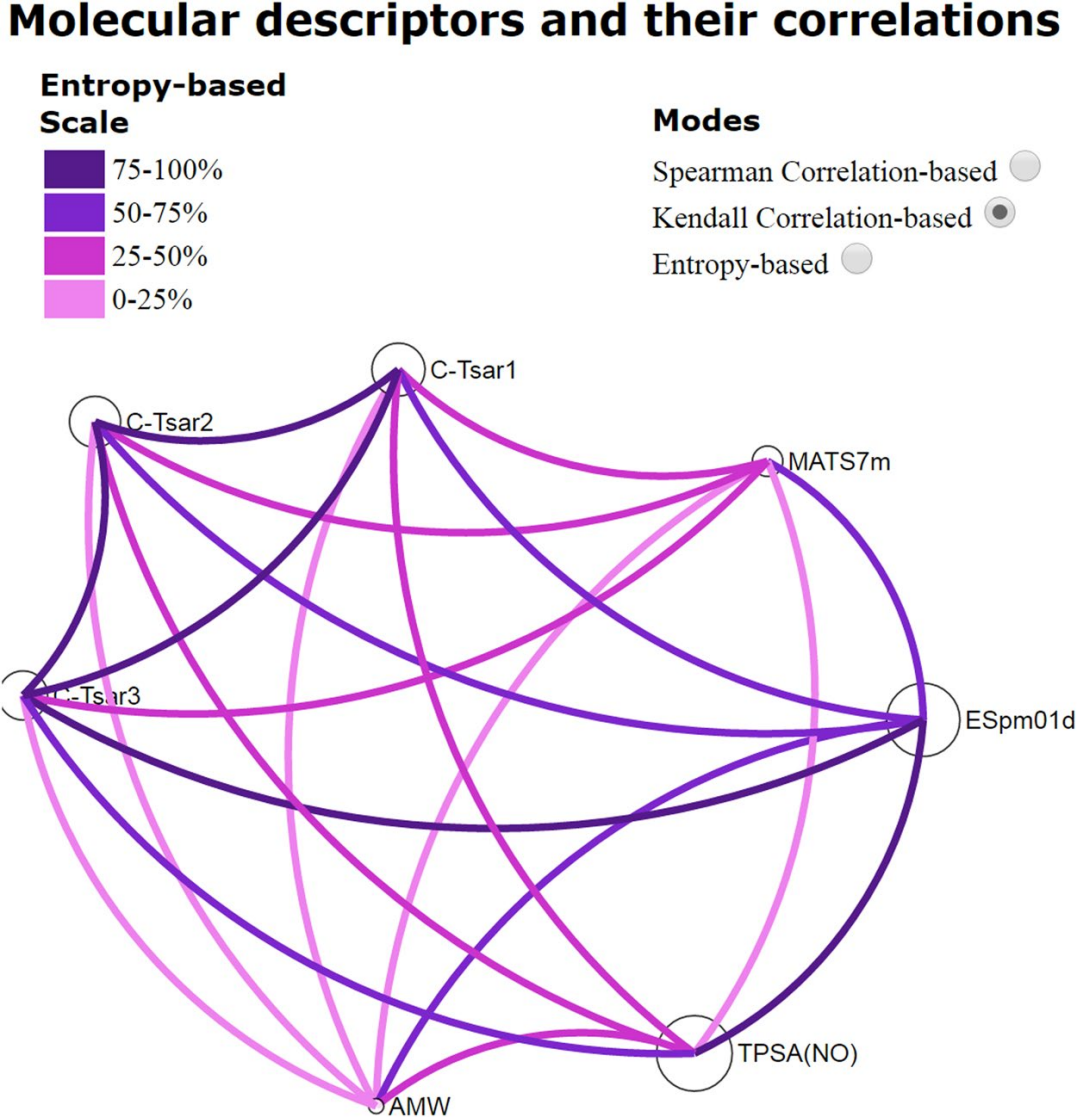
Mutual information analysis among the molecular descriptors that conform the M5_HIA_ ∪ CT_HIA_ model.

For human intestinal absorption (HIA), regression and classification QSAR models with higher performances were obtained from different molecular descriptor subsets. The best regression QSAR model was obtained using a combined subset of descriptors (M5_HIA_ ∪ CT_HIA_). M5_HIA_ subset is integrated by four molecular descriptors that correspond to different families. Molecular weight has been found as a key parameter also in this model, in concordance with the Lipinski rule that establishes, among other properties, an MW <500 Da to obtain orally active compounds. Also, topological polar surface area (TPSA) corresponding to molecular properties family has been found as one of the key descriptors in the best model. In the same sense, it is well-known the correlation between the polar surface area of molecules and their capacity to undergo human intestinal absorption. There are also two more descriptors spectral moment 01 from edge adjacency matrix weighted by dipole moments (ESpm01d) and MATS7m (Moran autocorrelation of lag 7 weighted by mass). The former makes use of spectral moments of the edge weighted adjacency matrix. This approach is a structure-explicit scheme which uses a well-defined mathematical invariant and has a direct interpretation in terms of structural fragments of the molecules having some resemblance with the additive schemes of Free-Wilson and Fujita-Ban. The last descriptor is a Moran autocorrelation of lag 7 regarding molecular weight. On the other hand, we can use VIDEAN in the same way that BBB in order to analyze the relationship among the descriptors provided for M5_HIA_ and CT_HIA_ in M5_HIA_ ∪ CT_HIA._ In this case, Fig. 5 shows mutual information obtained by Entropy-based mode. This mode has a single scale (%) where it is desirable a low entropy (light pink) between descriptors (low mutual information). As it can be seen, in Fig. 5 CODES-TSAR descriptor edges are purple meaning high mutual information, but each one (CODES-T1, CODES-T2 and CODES-T3) presents low entropy with the other M5_HIA_ descriptors (light pink and pink). This fact demonstrates the complementary information provided by both subsets, M5_HIA_ and CT_HIA_.

The best classification QSAR model was obtained using the M9_HIA_ subset. This model achieves the highest accuracy (86.96%) and an elevated average ROC area (0.865). For the HIA classification experiments, two classes were defined: not absorbed molecules and absorbed molecules (see discretization thresholds in Materials and Methods section). From the confusion matrix, we can observe that this classifier has a high precision for absorbed compounds (92.30%) and a more moderate precision for not absorbed compounds (70%). The performance decay for the second class can be related to the class imbalance in the testing set because only the 25% of the samples corresponds to molecules which are not absorbed.

**Figure 6:**
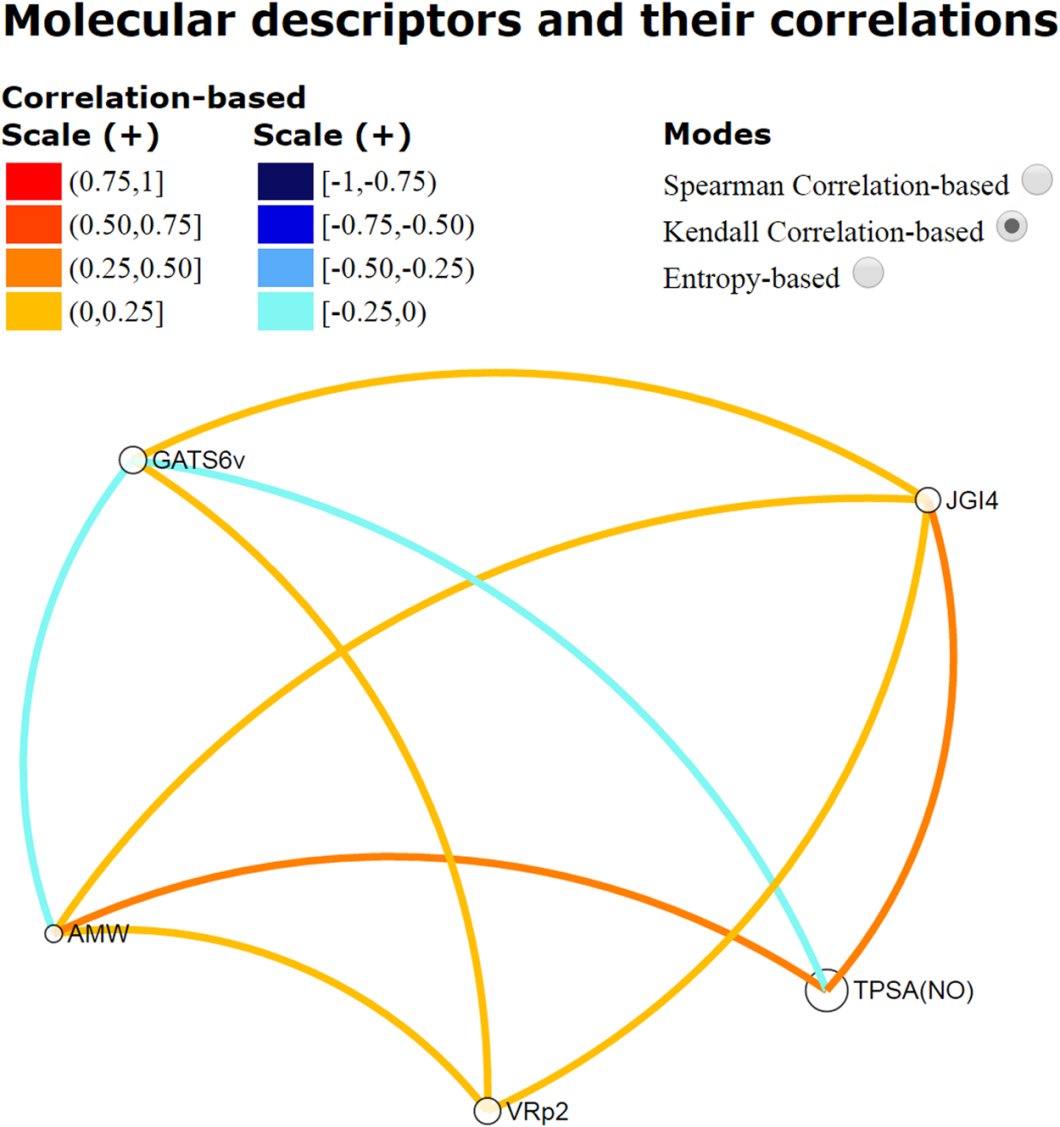
Correlation analysis among the molecular descriptors that conforms the model M9_HIA_.

M9_HIA_ presents a total of five molecular descriptors, two of which are also involved in the previous regression model, AMW and TPSA, thus strengthening the important correlation of these molecular properties with HIA values. Another descriptor found in the model is GATS6v (Geary autocorrelation of lag 6 weighted by van der Waals volume), a general index of spatial autocorrelation regarding van der Waals volume in this case. Geary coefficient is a distance-type function varying from 0 to infinite. Strong autocorrelation produces low values of this index; moreover, positive autocorrelation translates into values between 0 and 1 whereas negative autocorrelation produces values larger than 1; therefore, the no correlation reference is 1. Besides the GATS6v, there is another descriptor in the model belonging to the 2D autocorrelation family, the JGI4 (mean topological charge index of order 4). This descriptor is able to evaluate the charge transfer between pairs of atoms, and therefore the global charge transfer in the molecule. Finally, the last descriptor for this model is the VRp2 (average Randic-type eigenvector-based index from polarizability weighted distance matrix), an eigenvalue-based index. As before, we analyzed the M9_HIA_ descriptors relationship in correlation terms by using VIDEAN. In Fig. 6, the graph for Spearman-based mode can be seen. It can be observed that a low correlation between descriptors is represented by light tones of colors: light orange and light blue. Once more, this result is the desirable one, where each descriptor provides singular information to the model.

In summary, the two DELPHOS subsets used for regression and classification, M5HIA and M9HIA, combine several descriptors from different families, where the most populated family is the 2D autocorrelation including Geary and Moran autocorrelation regarding van der Waals volume and mass respectively, as well as topological charge. As it was previously mentioned, there are 2 descriptors present in both models that have been widely used to predict oral bioavailability. There are studies regarding classification and regression trees^25^ as well as very good correlations^26^ using PSA for HIA prediction. It is also widely accepted that molecules with PSA lower than 60 exhibit high and almost complete intestinal absorbance, whereas molecules with PSA higher than 140 reveal poor intestinal absorbance. Therefore, the physicochemical relevance of the molecular descriptors selected by DELPHOS is well-supported by previous evidence.

#### Statistical significance of CT_HIA_ descriptors contribution to the combined model M5_HIA_  ∪  CT_HIA_

**Figure 7:**
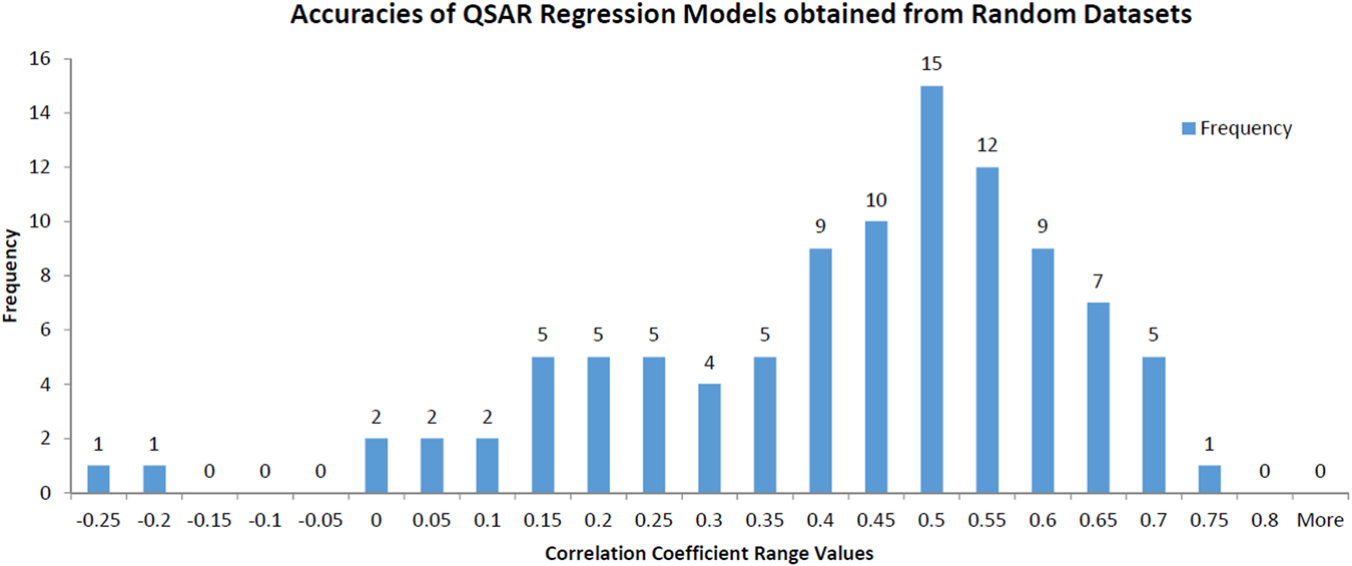
Accuracy frequency values for QSAR regression models computed using random combined subsets of descriptors.

Regarding the actual contribution of the CT_HIA_ descriptors to the accuracy of the combined regression QSAR model, we decided to execute an additional experiment. The idea was to evaluate the statistical significance of CT_HIA_ descriptors contribution to the combined model in contrast with a random selection of molecular descriptors. For this analysis, one hundred replicates of a random experiment were executed. In each replicate, three molecular descriptors included in CT_HIA_ are substituted by three molecular descriptors (RAND_HIA_) randomly taken from the entire set of molecular descriptors computed by DRAGON (excluding the molecular descriptors of M5_HIA_). After this replacement, a regression QSAR model is recomputed using the new combined subset M5_HIA_ + RAND_HIA_, applying the same experimental conditions reported for the best HIA QSAR regression model (see Table 1). In this way, a frequency distribution of correlation coefficients (CC) values is obtained from one hundred QSAR models inferred with these random subsets, which is depicted in Fig. 7. Analyzing these results, we can observe that the mean value of accuracy of these QSAR models is very low (0.397). Moreover, no regression model generated from the random subsets achieved the correlation coefficient of the best regression model for HIA (0.76). Therefore, we can conclude that the contribution of CT_HIA_ subset to the combined model is clearly relevant in statistical terms.

In summary, we can assert that best QSAR model obtained for HIA dataset by combining DELPHOS with CODES-TSAR achieves reasonable prediction accuracies. The molecular descriptors chosen by the feature selection method are clearly related with the HIA in physicochemical terms, and they also present low redundancy levels. For the particular case of the combined regression model, we can observe that molecular descriptors provided by CODES-TSAR improved the performance of QSAR model generated by DRAGON/DELPHOS with statistical significance. Beyond the analysis of the best QSAR models, from the complete set of experiments reported on the Supplementary Table S4, we computed confidence intervals in order to determine if there are differences with statistical significance in the performance of both approaches. The results reveal that DRAGON/DELPHOS approach outperforms CODES-TSAR with statistical significance in both QSAR modeling strategies, regression and classification, for this dataset.

### QSAR models for enantiomeric excess (EE)

#### Drug-like properties calculation and similarity assessment

Asymmetric catalysis has generally been accepted as the best methodology for the preparation of enantiomeric compounds and consequently is one of the most prominent and competitive area of research in current organic synthesis. The spectacular progress achieved in this area in last decades has been underpinned by the development of a plethora of efficient chiral ligands, many of them now commercially available. However, their effectiveness strongly depends on the structure of the substrate; therefore, the vast diversity of organic compounds has hampered the development of catalysts operative for a broad spectrum of transformations. In this context, a considerable experimental effort has to be devoted to finding the appropriate chiral catalyst for the desired reaction. We envisage that computational methods can help to predict the enantiomeric excess for a defined set of catalysts and reactants, and hence this opens a new approach to estimate the best chiral ligand for a desired reaction without the need to perform the reaction^27^.

The palladium(0)-catalyzed asymmetric allylic substitutions (Tsuji-Trost reaction) is one of the most powerful procedures for the enantiocontrolled formation of carbon-carbon or carbon-heteroatom bonds^28–33^. Since the first examples in the early seventies, a vast number of very efficient chiral ligand has been developed for this transformation. In particular, the asymmetric allylic substitution of allylic acetates or benzoates with dialkyl malonate has been extensively used as a successful test bench for the design and development of new chiral ligands. Given the large amount of literature data for this reaction, in which a vast array of chiral ligands of different backbone, coordination atoms and coordination modes has been tested, we have selected this asymmetric transformation as a model reaction to test the viability of our initial hypothesis.

In the study of QSAR models for enantiomeric excess, we have collected a dataset of 177 ligands and 9 substrates (see Supplementary Zipped File 3) with known experimental values of *ee* from the literature. Considering this data, ligands, substrates, and enantiomeric excess values were correlated.

To measure the structural diversity of this dataset, we have carried out studies in an analogous way as with the previous databases. Only ligands were considered to develop the QSAR studies, as substrates show a high similarity among them in terms of descriptors. Therefore, using CODES-TSAR descriptors, we have performed a similarity analysis by the mean Pearson Similarity index, and the data was ordered in an increasing way from 0 to 1. The similarity indices for EE dataset using CODES-TSAR descriptors are shown in Supplementary Table S5. As it can be observed, the compounds in the dataset present a wide range of diversity from total dissimilarity to similarity for a reference compound. This fact may allow us to foresee that the applicability domain of the models built in this study will be wide as in the previous models.

**Figure 8:**
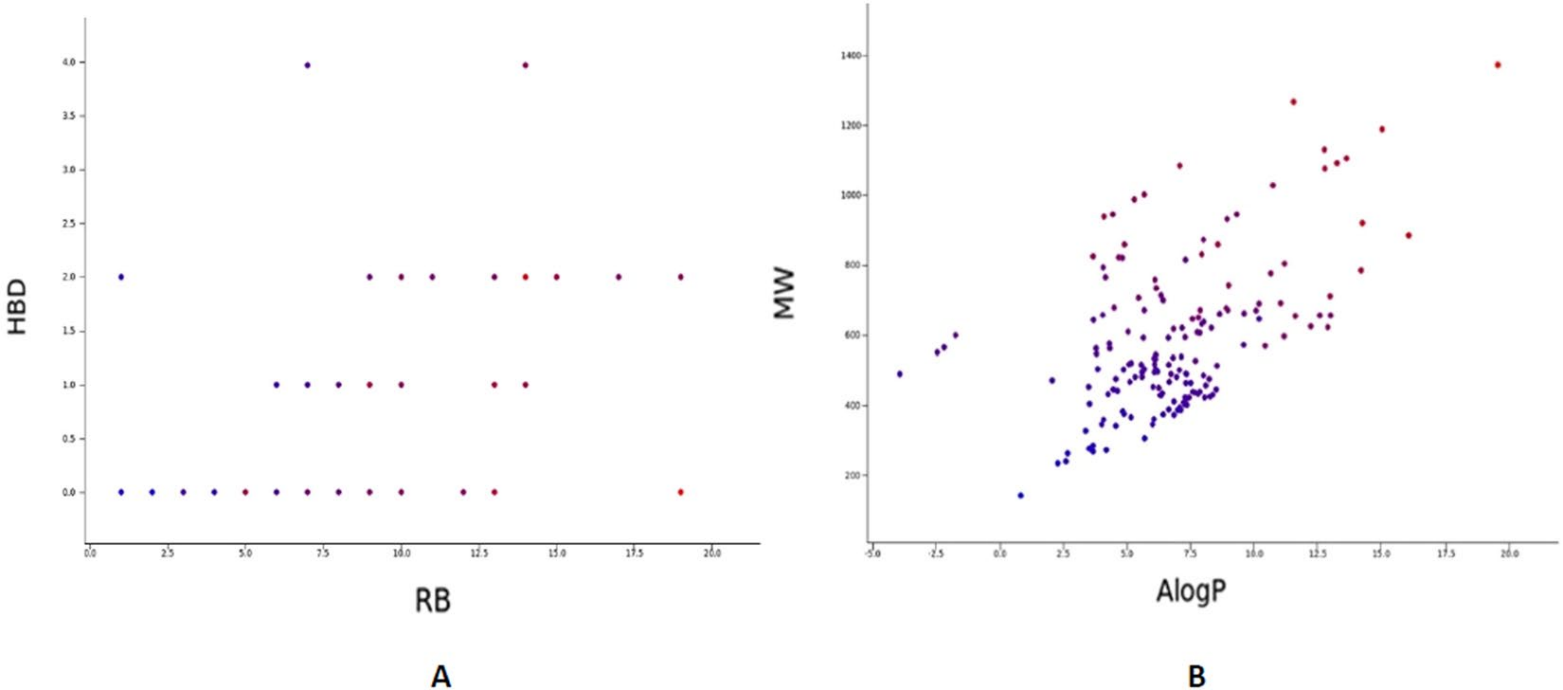
Physicochemical representation of the EE dataset. (**A**) Dispersion of the compounds regarding hydrogen bond donors and rotatable bonds (RB). Color is defined by molecular weight (MW). (**B**) Dispersion of the dataset taking into account logP values and molecular weight.

Due to the structural complexity of this dataset, that contains ligands with metal complexes on their backbone (e.g., a ferrocene backbone), physical-chemical properties were not able to be calculated using Qikprop module. However, Canvas module^34–36^ allows us to calculate molecular properties for this dataset. The most representative descriptors given by the software were analyzed in order to show the diversity of the dataset, as it is depicted in Fig. 8, in which parameters such as Molecular Weight (MW), Hydrogen bond acceptors and donors (HBA and HBD respectively) or polar surface area (PSA) are some of the descriptors plotted. The data shows a wide distribution of the physicochemical properties: regarding MW values, compounds are in a range between 142 and 1372, HBA are in the interval from 0 to 19, while rotatable bonds are in the range between 1 and 19. From this analysis it is possible to extract two central conclusions: the first one, this dataset presents a wide range of diversity, an important feature for QSAR models. The second one is related with the low level of drug-like properties of this dataset in comparison with both previous datasets. This fact is due to the higher values of logP, showing most of the compounds values between 5 and 15 and MW with values reaching 800 and even 1000 Da.

#### Molecular Descriptor Subsets selection and QSAR models inference

For this dataset, we have again decided to pick-up the two subsets with the lowest RAE values reported by DELPHOS, which correspond to M9_EE_ and M14_EE_ subsets. Therefore, for the EE dataset, five alternative subsets of molecular descriptors were used for the experiments: one subset obtained from CODES-TSAR (CT_EE_), two subsets selected by DRAGON-DELPHOS (M9_EE_ and M14_EE_) and the union of M9_EE_ and M14_EE_ subsets with CT_EE_ (M9_EE_ ∪ CT_EE_ and M14_EE_ ∪ CT_EE_). Table 4 summarizes the characteristics of these subsets. It is important to note that molecular descriptors for substrate and ligand databases were selected by DELPHOS for each subset. The texts “Sust” and “Lig” were concatenated to the DRAGON descriptor names in order to identify from which database they were selected. Regarding CODES-TSAR subset, four descriptors were extracted from substrates (Sa, Sb, Sc, and Sd) and six descriptors were extracted for ligands (La, Lb, Lc, Ld, Le, Lf).

**Table 4:** Molecular descriptor subsets used for inferring the QSAR models for enantiomeric excess.

Subset Name	Method	Size	Molecular descriptor names
CT_EE_	CODES-TSAR	10	Sa, Sb, Sc, Sd, La, Lb, Lc, Ld, Le, Lf
M9_EE_	D/DELPHOS	4	AMW Sust, EEig11d Sust, JGI6 Sust, TPSA.NO. Lig
M14_EE_	D/DELPHOS	17	AMW Sust, PJI2 Sust, EEig12x Sust, EEig09d Sust, EEig11d Sust, GGI8 Sust, nDB Lig, nH Lig, nR09 Lig, TI2 Lig, PW5 Lig, D.Dr08 Lig, AAC Lig, MATS5v Lig, MATS8v Lig, MATS3p Lig, GATS1e Lig
M9_EE_ ∪ CT_EE_	Combined	14	AMW Sust, EEig11d Sust, JGI6 Sust, TPSA.NO. Lig, Sa, Sb, Sc, Sd, La, Lb, Lc, Ld, Le, Lf
M14_EE_ ∪ CT_EE_	Combined	27	AMW Sust, PJI2 Sust, EEig12x Sust, EEig09d Sust, EEig11d Sust, GGI8 Sust, nDB Lig, nH Lig, nR09 Lig, TI2 Lig, PW5 Lig, D.Dr08 Lig, AAC Lig, MATS5v Lig, MATS8v Lig, MATS3p Lig, GATS1e Lig, Sa, Sb, Sc, Sd, La, Lb, Lc, Ld, Le, Lf

Using these descriptor subsets, several regression and classification QSAR models were inferred applying different machine learning approaches. The accuracy metric values achieved per each QSAR model are reported in Supplementary Table S6 and all CSV files used for running the EE experiments in WEKA are made available as Supplementary Zipped File 4. The regression and classification QSAR models with better performance were obtained using the CT_EE_ subset learned by CODES-TSAR, which is formed by 4 substrate descriptors and 6 ligand ones, final size equaling 10. In particular, the best classification model obtained from the CT_EE_ subset achieves a high level of accuracy (84.38%). For the EE classification experiments, two classes were defined: low-enantiopurity (0–90% ee) and high-enantiopurity samples (90 -> 99% ee, see discretization thresholds in Materials and Methods section). From the confusion matrix, we can observe that this QSAR model has a high precision for low-enantiopurity samples (94.94%) and a poor precision for high-enantiopurity samples (35.29%). The weak performance obtained for the second class can be related to the strong class imbalance in the testing set, where only the 17.71% of samples corresponds to a pure class. This fact can also explain the low value of the average ROC area (0.588).

**Figure 9:**
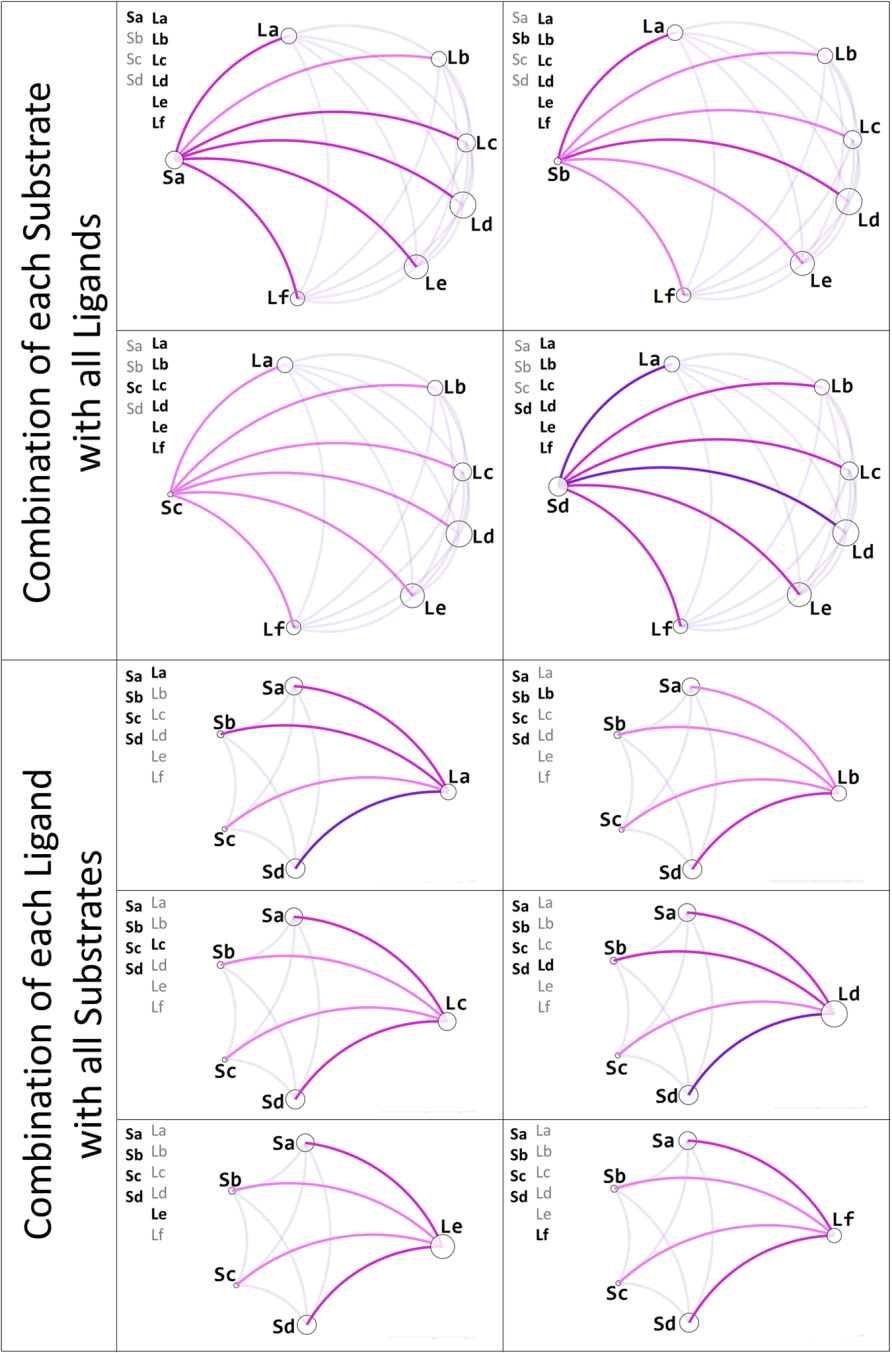
Mutual information among the substrate and ligand descriptors computed by CODES-TSAR (CT_EE_ subset).

**Figure 10:**
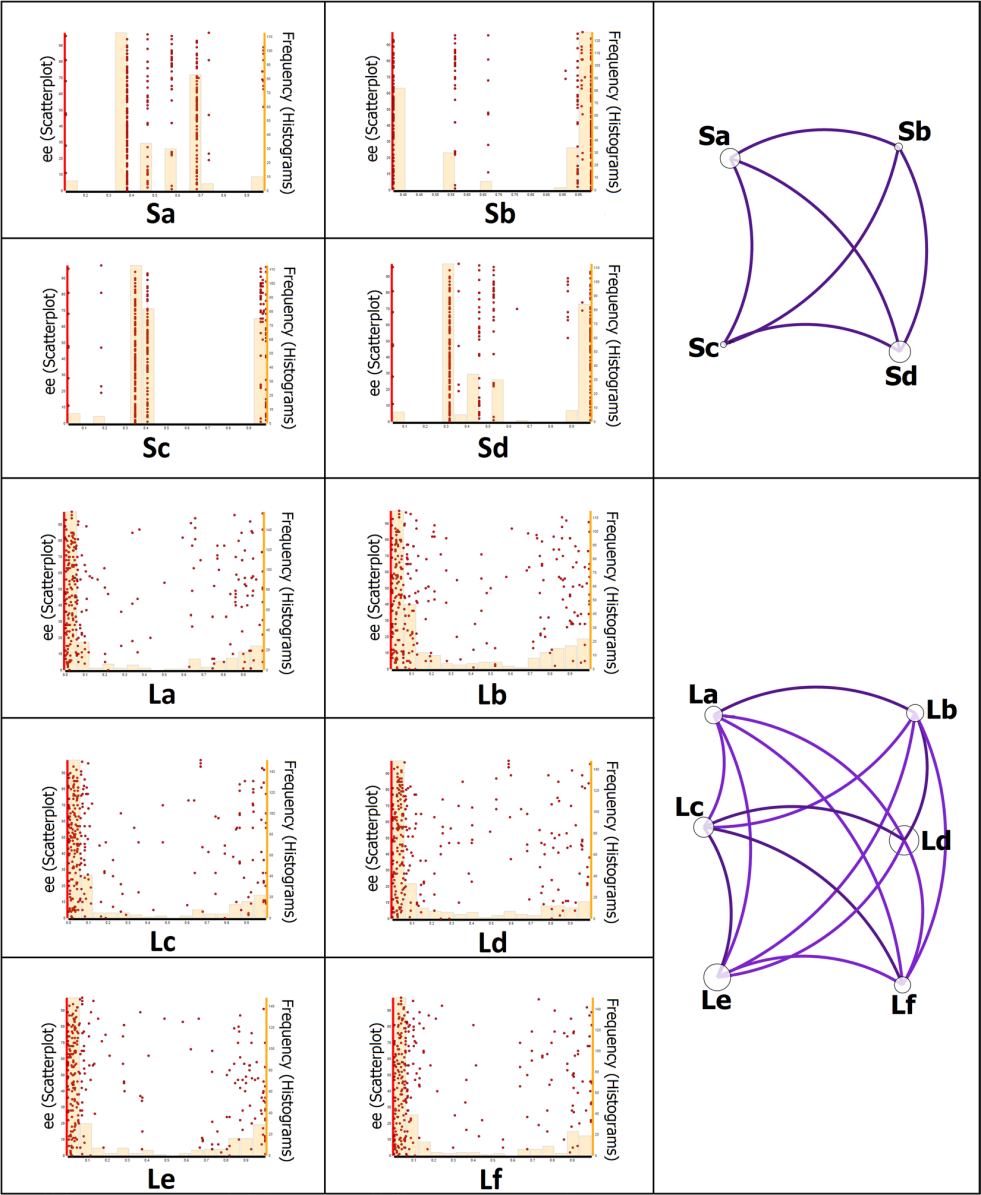
Relationship among the molecular descriptors computed by CODES-TSAR (**CT**_**EE**_ subset) and the target property.

A physicochemical interpretation is not possible to be made because of the nature of CODES-TSAR technique (See Introduction). Consequently, we only analyze the descriptor relationships in statistical terms by using VIDEAN. In Fig. 9, the entropy-based mode is shown for relationships among substrate and ligand descriptors, considering one at a time. In general terms, all the edges are light pink and pink, demonstrating low mutual information and consequently good complementary. Additionally, in Fig. 10, we can see the analysis for both groups of descriptors (substrate and ligand), where all edges are purple. This behavior is not relevant to the group, because of the nature of CODES-TSAR technique (See Introduction), but the combination of groups shown in Fig. 9 is indeed important.

Furthermore, another functionality offered by VIDEAN is the visualization of scatter plots and their associated histograms. The goal is to see the behavior of descriptor values versus the target property, in order to realize how the information zone is covered by different descriptors. In Fig. 10, this explanation is more deeply understood. It can be seen a 10 scatter plot with its related histogram, one for each model descriptor. The analysis can be made in two groups, first for substrate descriptors and then for the ligand ones. Sa, Sb, Sc, and Sd mostly show values in the median and right zone (see histograms). On the other hand, La, Lb, Lc, Ld, Le, and Lf show values in the left zone (see histograms), where substrate descriptor does not present any value. Consequently, we can infer that the combination of the two groups is completing the information zone for the model, and this coverage is desirable for QSAR modeling.

In summary, we can assert that, for EE dataset, the best QSAR models obtained by CODES-TSAR achieve reasonable prediction accuracies. The molecular descriptors chosen by the feature learning method have low redundancy levels. Besides, we can observe that the combination of two groups of molecular descriptors (substrates and ligands descriptors) provided by CODES-TSAR achieves a complete coverage of the information zone for the QSAR model. Beyond the analysis of the best QSAR models, from the complete set of experiments reported on the Supplementary Table S6, we computed confidence intervals in order to determine if there are differences with statistical significance in the performance of both approaches. For this dataset, the results reveal that CODES-TSAR approach outperforms DRAGON/DELPHOS in average for both QSAR modeling strategies, regression and classification. Nevertheless, these differences have not statistical significance.

### Impact of the Hybridization Approach

As it was mentioned before, a relevant goal of this work is to assess the potential benefits related to the hybridization of feature selection and feature learning approaches in QSAR modeling. More specifically, the aim is to determine the potential benefits of combining molecular descriptor subsets computed by DRAGON-DELPHOS (D − D) and CODES-TSAR (C − T) methodologies for the inference of regression and classification QSAR models in drug design.

**Figure 11:**
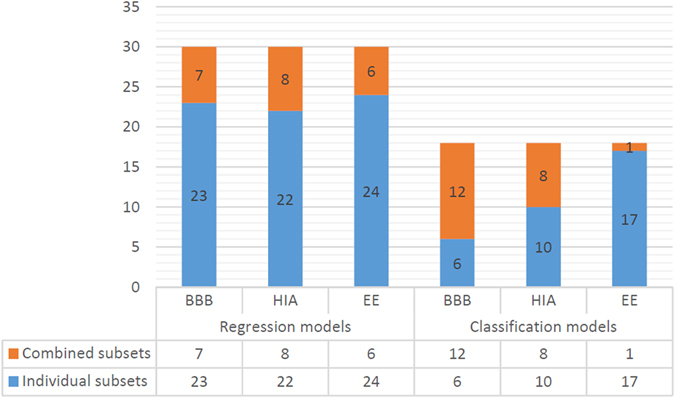
Number of experimental scenarios where QSAR models obtained by combined subsets improve the performance of the QSAR models inferred by individual subsets.

Analyzing all experiments executed for each dataset under different experimental conditions (combinations of different molecular descriptor subsets, machine learning methods, and sampling sizes, see Fig. 1), a total of thirty and eighteen different experimental sceneries can be defined for regression and classification experiments respectively (see Supplementary Tables S2, S4 y S6). Figure 11 shows the proportion of sceneries were QSAR models inferred by combining molecular descriptor subsets of both approaches outperform the accuracy of the QSAR models inferred for D − D and C − T subsets alone. From this chart, it is clear that regression models inferred from the individual subsets have, in general, better accuracy that the combined ones. Only for the classification models inferred from the BBB dataset, it is observed that combined subsets outperform the individual subsets in most of the experimental scenarios. These results allow us to conclude that the hybridization of both strategies (feature selection, and feature learning) can be useful but the performance depends on the dataset characteristics.

### Impact of the WEKA training method used for inferring the QSAR models

Another factor of relevance for the practitioner is how to choice the methodology used for the inference of the QSAR models. In this paper, we explore the use of different training methods provided for WEKA tool for regression and classification problems. Therefore, from the whole set of experiments generates in this work (see Supplementary Tables S2,S4 y S6), confidence intervals of the performance differences between each pair of training method were computed. In both cases, regression and classification models, Random Forest and Random Committee methods achieved a better accuracy that the other methods (Neural Networks, Decision Trees and Linear Regression) with statistical significance. Nevertheless, the differences between Random Forest and Random Committee are negligibly in both scenarios (regression and classification). For this reason, our piece of advice for the practitioners is the use of training methods based on ensembles, like Random Forest and Random Committee are, because their accuracies outperform the most traditional machine learning methods.

## Discussion

During the last decades, several feature selection and feature learning methods have been applied to the inference of molecular descriptor subsets for QSAR modeling. These models play a central role in the virtual screening of drugs, allowing the study of relevant physicochemical properties even before the synthesis of newly designed compounds. In particular, two machine learning methodologies, DRAGON-DELPHOS and CODES-TSAR, as representatives of these two approaches, have been compared in this work. The experiments were carried out with compound datasets for QSAR modeling of three different issues: blood-brain-barrier, human intestinal absorption, and enantiomeric excess.

Each dataset used during the machine learning experiments was characterized in detail by drug-like properties calculation and similarity assessment of their molecular descriptors. In all cases, QSAR model performances were contrasted for several experimental conditions, varying sampling parameters and techniques used for inferring the classification and regression models. The molecular descriptor subsets obtained by DRAGON-DELPHOS and CODES-TSAR strategies were also analyzed in terms of mutual information and correlation, in order to evaluate the pairwise associations among the relevant descriptors and their relationships with the properties under study. From the results, we observed that none of the methods outperform the other one in all scenarios since the prediction accuracy depends on database features and experimental conditions. Nevertheless, regarding the training methods used for QSAR model inference, the techniques based on ensembles, Random Forest and Random Committee, outperform with statistical significance the most traditional algorithms in the two kind of QSAR models (regression and classification). For this reason, we recommend to practitioners to apply ensemble based methods for the model training step.

Another piece of advice for QSAR modelers is associated with the intrinsic characteristics of each methodology. CODES considers that the property to study depends on chemical structure of the molecule, and not a contribution of different independent variables. In fact, CODES codifies a structure (generation of a small set of descriptor) from the chemical structure of the molecule based on the atom nature, the number of atom bonds and the connectivity with the rest of the molecule. Therefore, with this tool is not necessary to compute a selection of features step like DELPHOS does. In the other hand, each feature calculated by DRAGON has its own physicochemical interpretation and can be used in a QSAR model as an individual piece of information. Therefore, the interpretation of QSAR models in terms of the individual contribution of the molecular descriptors is possible, helping to obtain more understandable models. For this reason, each modeler can chose a methodology taking into account in which aspect is focused: computational efforts or model interpretability.

Beyond the use of these feature identification approaches separately, as alternative competing methodologies, in this study we also decided to assess the impact of hybridizing both techniques. This decision was based on recent results, published in the area of QSAR modeling for material design, where the combination of both methods improved the prediction quality. These hybridization experiments for our datasets reveal that QSAR models accuracy can be enhanced by joining molecular descriptor subsets obtained by both methodologies if these subsets contain complementary information for the models, such as it occurred with the best HIA regression model. For this reason, as a general conclusion, we recommend to the virtual screening practitioners to consider this hybridizing philosophy as an additional strategy for their experiments.

It also interesting to remark that QSAR classifiers with a high accuracy percentile (around 85%) were obtained from the three datasets. Nevertheless, in all cases, different degrees of unbalance among the number of samples available for each class in the testing sets affected the average ROC area values. Therefore, even when this paper is focused on the comparison between two feature selection and feature extraction methods, together with their potential hybridization, we hope to enhance the classifiers by applying techniques for artificial balancing of sample classes in forthcoming experiments. Finally, it is possible to hybridize another alternatives methods for feature selection and feature learning as future work.

## Materials and Methods

### Preparation of Databases

#### Ligand preparation

The HIA and BBB datasets on SMILES format were converted to 3D structures using LigPrep1 software implemented on Maestro Suite2^37^. LigPrep^38^ is a 2D-to-3D conversion tool that includes the addition of hydrogen atoms and options for generating multiple possible tautomers, stereoisomers, ionization at a selected pH range, and ring conformations using molecular mechanics force fields. To carry out our studies, possible ionizations were generated at pH 7.3 in order to obtain the most suitable ionization states of the compounds for that pH range. The ionization states were assigned with Epik3 module^39^. Also, all the compounds were desalted and no tautomers were generated. In this process, we have restricted the search to obtain just one possible stereoisomer among all that can be found by the program, as well as one low energy ring conformation. The final step of a LigPrep preparation is an energy minimization of the 3D conformers generated using the OPLS 2005 force field^40^.

Different conformers and ionization states of the same compounds were reduced in order to keep one 3D structure per initial compound. The selection was made considering the most probable ionization state at physiological pH conditions. This preparation is a crucial step for the following studies and was performed with the aim of obtaining the most suitable 3D structures to further calculate the physicochemical properties of the existing compounds.

#### Drug-like properties calculation

All the prepared compounds were analyzed using Qikprop^26^ module of the Small-Molecule Drug Discovery Suite in Schrödinger, an accurate and quick software that predicts structurally significant 2D and 3D descriptors and pharmaceutically relevant properties of organic molecules. Absorption, Distribution, Metabolism, and Excretion (ADME) properties were predicted using the program QikProp where a total of 44 properties could be predicted. Among all the properties, the program calculates properties like molecular weight, molecular volume, number of H-bond donors, number of H-bond acceptors, polar surface area, QPlogPo/w (predicted octanol/water partition coefficient) and violations related to the Lipinski’s Rule of 5 and Jorgensen’s Rule of 3, to filter out compounds with clear cut undesirable properties for drug discovery. For the EE dataset, and due to chemical structures of the dataset that contains coordination complex, Canvas^41^ software was used. This tool is a cheminformatics package that provides a range of applications for structural and data analysis, including fingerprints, similarity searching, substructure searching, selection by diversity, clustering, building regression and classification models. In this case, it allowed us to calculate physicochemical properties in an analogous way that Qikprop.

#### Similarity calculations

The similarity indices were calculated using CODES parameters, three for each compound of HIA and BBB datasets and 6 parameters to EE dataset. Similarity calculations for the three datasets were performed using the SPSS software^41^. Distances were computed between cases measuring similarities by Pearson correlation. The values were transformed into a standardized range of 0 to 1 by variable, and the measures were transformed and rescaled to a 0–1 range. With these parameters, the similarity was computed for all three databases thus obtaining different correlations between the compounds. For every dataset, one compound was chosen to be the reference and similarity is described for the rest of the datasets.

Also, a representation of CODES descriptors was performed using SigmaPlot as a measure of structural diversity.

### Software used for Processing Molecular Descriptors

The first step before applying a feature selection method consists in calculating the molecular descriptors. This task is performed in this paper with DRAGON software^8^. DRAGON is an application tool for the calculation of molecular descriptors. It provides almost 5,000 molecular descriptors (0D, 1D, 2D, and 3D), which can be used to evaluate molecular structure-activity or structure-property relationships of molecule databases. To calculate these molecular descriptors, molecular structure files are required. DRAGON can also deal with H-depleted molecules and 2D-structures. Then, the stage that takes place is the selection of features and it is performed with DELPHOS^11^.

In the other hand, for feature learning method, the first step is carried out by CODES. CODES is a software based on artificial neural computing. It generates descriptors correlated with the atom nature, the atom bonds and the connectivity with the rest of the molecule. In fact, each point (atom) of the topological space corresponds to each unit (neuron) of the neural space, and each binary relation (bond) corresponds to each connection of the neural space. This results in a neural network designed as an interactive activation and competition network, which is processed until an equilibrium state is reached^12^. The next step consists in the reduction of the dimension of matrices of each compound. TSAR is the software responsible for the dimension reduction process. Reduction of dimension philosophy resides in reducing the complexity of any system without loss information. This process is achieved by training a supervised multilayer neural network namely ReNDer (Reversible Non-linear Dimension reduction). TSAR program applies a Monte Carlo algorithm and the same number of descriptors for all molecules in databases was obtained^18^.

### WEKA Machine Learning Methods used for Inferring Regression and Classification Models

Weka is a collection of machine learning algorithms for data mining tasks^20^. The methods used in this study are described next:

Linear Regression: Class for using linear regression for prediction. Uses the Akaike criterion for model selection, and is able to deal with weighted instances.

Decision Tress: Classifier for building and using a decision stump. Usually used in conjunction with a boosting algorithm. Does regression (based on mean-squared error) or classification (based on entropy). Missing is treated as a separate value.

Neural Networks (Multiperceptron): A Classifier that uses back-propagation to classify instances. This network can be built by hand, created by an algorithm or both. The network can also be monitored and modified during training time. The nodes in this network are all sigmoid (except for when the class is numeric in which case the output nodes become unthresholded linear units).

Random Forest: Class for constructing a forest of random trees^42^. The random trees for constructing a tree that considers K randomly chosen attributes at each node. Performs no pruning. Also, it has an option to allow estimation of class probabilities (or target mean in the regression case) based on a hold-out set (back fitting).

Random Committee: Class for building an ensemble of randomized base classifiers. Each base classifier is built using a different random number seed (but based on the same data). The final prediction is a straight average of the predictions generated by the individual base classifiers.

Table 5 shows discretization criteria for target properties with their thresholds and explication. For HIA models the threshold under 0.7 is considered not absorb, while a threshold equal or above 0.7 is considered that absorb. The BBB model has three classes, molecules which cross the BBB (BBB+), molecules that do not cross the BBB (BBB-) and a gray area that represents uncertainty. The EE model has two tags, low-enantiopurity in the center zone from 10% to 90% and high-enantiopurity in the extremes from 0% to 10% and from 90% to 100%.

**Table 5:** Discretization criteria for target properties.

HIA	Tag	Not Absorb	Absorb	
	Thresholds	<0.7	>=0.7	
BBB	Tag	BBB+	Gray area	BBB−
	Thresholds	<=−0.7	>−0.7 y < = −0.3	>−0.3
EE	Tag	Low-enantiopurity	High-enantiopurity	
	Thresholds	from 10% to 90%	from 0% to 10%	
			from 90% to 100%	

### CODES software

CODES® program is able to encode a molecule from a topological point of view into molecular descriptors in which all the underlying information of the chemical structure is contained. Although CODES was initially designed for MAC computers, a new version for Windows XP with graphical interface has been developed in collaboration with Advanced Software Production Line, S.L. This new version allows the reading of the structure through its SMILES code into a graphical interface.

**Figure 12:**
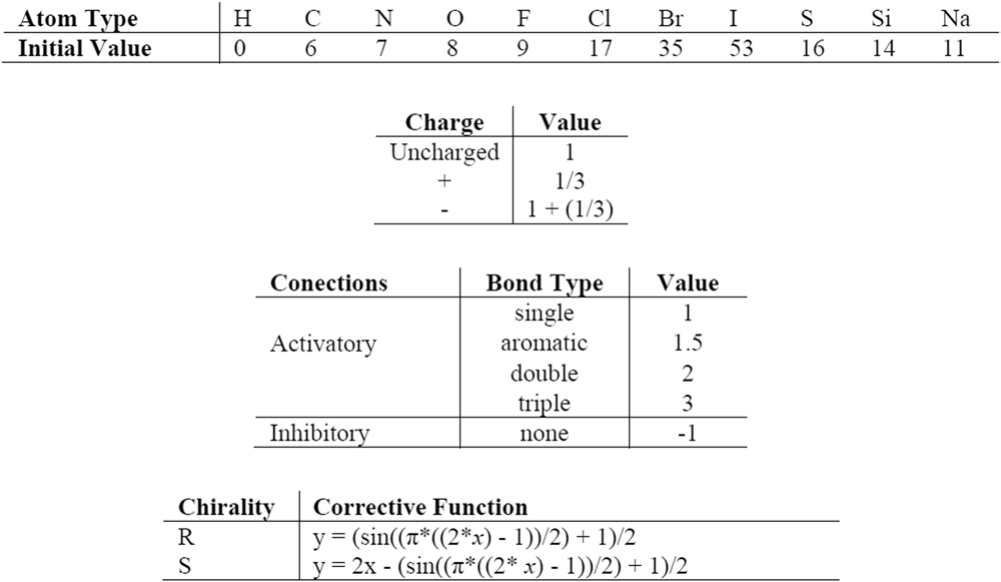
Values and functions assigned by CODES.

CODES consists of two levels, a topological and a neural one, and its philosophy lies in a Gestalt isomorphism between both levels. While the topological space is the chemical structure in itself, the neural one consists in an interactive and competitive network. Each point or atom of the topological space corresponds with each unit or neuron of the neural space, and each type of atom takes a different initial value. Attending to connectivity, CODES considers both bonded and not bonded atoms. If atoms are not bonded in the topological space, it means an inhibitory connection in neural level, otherwise, the neural space considers an excitatory connection and the value depends on bond type. The stereochemistry is also taken into account during the codification process and R or S chirality is expressed by a corrective non-linear function (Fig. 12).

The neural network employs a sigmoideal function in the codification process and the network is characterised by a non-supervised learning. In the learning process, CODES records all the activities reached in every iteration of the network and it is processed until an equilibrium state is reached, so that we have a set of temporal values, cast into a matrix of AxR dimensions, where *A* is the number of atoms included in the SMILES code and *R* is the number of iterations that CODES function needs to achieve this equilibrium stage. In fact, this is a dynamical matrix of descriptors because takes into account the whole codification progress. We have also the chance to choose only the last step of codification, so we would have a static set of descriptor of the molecules but, in order to perform a compression of the information without loss of any of the calculated descriptors, we have selected the matrix with the whole codification progress.

*Reduction of Dimensions* (*RD*). The philosophy of this process resides in reducing the complexity of any system without loss of any intrinsic characteristics or information about the chemical nature. This process is carried out by a back-propagation neural network with architecture (AxR)-c-y-c-(AxR), where (*A*x*R*) represents CODES matrix, *c* is the number of neurons in codification layer and *y* is the number of hidden neurons. In the model developed, reduction of dimension process is carried out in order to compress the dynamic matrix data to a set of three numeric codes for each molecule (hidden neurons: X1, X2 and X3; see Supporting Information, Table S2). RD process is carried out using TSAR© program which applies Monte Carlo algorithm. Convergence parameters are 3000 iterations/cycle, 1000 cycles past best (it is the number of cycles that are completed without improving on the best RMS fit before the training is terminated) and convergence of 0.005 RMS (Root Mean Square). The neural network is considered trained when the convergence plot shows a constant behaviour.

## Electronic supplementary material

SupplementaryMaterial

Data Allylic Substitution Info

CTBBB classification

CTBBB regression

M13BBB+CTBBB classification

M13BBB+CTBBB regression

M13BBB classification

M13BBB regression

M2BBB+CTBBB classification

M2BBB+CTBBB regression

M2BBB classification

M2BBB regression

CTHIA classification

CTHIA regression

M5HIA+CTHIA classification

M5HIA+CTHIA regression

M5HIA classification

M5HIA regression

M9HIA+CTHIA classification

M9HIA+CTHIA regression

M9HIA classification

M9HIA regression

CTEE classification

CTEE regression

M14EE+CTEE classification

M14EE+CTEE regression

M14EE classification

M14EE regression

M9EE+CTEE classification

M9EE+CTEE regression

M9EE classification

M9EE regression
