# Supplementary material for: Hybridizing Feature Selection and Feature Learning Approaches in QSAR Modeling for Drug Discovery
Source: Sci Rep. 2017 May 25;7:2403. doi: 10.1038/s41598-017-02114-3 (PMC5445096; doi:10.1038/s41598-017-02114-3)
Supplement: Supplementary file 1 — SupplementaryMaterial [file 41598_2017_2114_MOESM1_ESM.doc]

**Hybridizing Feature Selection and Feature Learning Approaches in QSAR Modeling for Drug Discovery**

Ignacio Ponzoni, Victor Sebastian, Carlos Requena, Carlos Roca, María J. Martínez, Fiorella Cravero, Mónica F. Díaz, Juan A. Páez, Ramon Gomez Arrayas, Javier Adrio and Nuria E. Campillo

**Supplementary Material**

| 1.0000 | 2-Methylpentane |
| --- | --- |
| 0.9992 | o-Xylene |
| 0.9973 | Paraxanthine |
| 0.9960 | 3-Methylpentane |
| 0.9918 | 1,2,3,4-Tetrahydroquinoline |
| 0.9870 | 2,2-Dimethylbutane |
| 0.9845 | Cyclopropane |
| 0.9759 | Gentisic |
| 0.9731 | Hexane |
| 0.9684 | Ethylbenzene |
| 0.9608 | Fluroxene |
| 0.9508 | Verapamil |
| 0.9464 | Lupitidineb |
| 0.9256 | Clonidine |
| 0.8974 | Theobromine |
| 0.8796 | Haloperidol |
| 0.8633 | Sulforidazine |
| 0.8623 | Methylcyclopentane |
| 0.8507 | Desmethydesipramine |
| 0.8468 | Temelastineb,c |
| 0.8247 | Tacrine |
| 0.8143 | Tibolone |
| 0.8116 | 4-Hydroxymidazolam |
| 0.8091 | Flunitrazepam |
| 0.8066 | Quinidine |
| 0.8060 | Northioridazineb |
| 0.8060 | Clobazam |
| 0.8053 | Thioperamide |
| 0.8042 | Phenylbutazone |
| 0.8040 | Phenytoin |
| 0.8040 | Thiopental |
| 0.8040 | Chlorambucil |
| 0.8029 | Mepyramine |
| 0.8000 | Carbamazepine |
| 0.7961 | Desmethyldiazepam |
| 0.7959 | Trifluoroperazine |
| 0.7668 | Didanosine |
| 0.7559 | Methotrexate |
| 0.7446 | Thioridazinec |
| 0.7401 | Flumanezil |
| 0.7177 | Haloethane |
| 0.7098 | Midazolam |
| 0.7038 | Pergolide |
| 0.7005 | Diazepam |
| 0.6992 | Mirtazapine |
| 0.6956 | Methohexital |
| 0.6952 | Levodopa |
| 0.6943 | Desmonomethylpromazine |
| 0.6942 | Aminopyrine |
| 0.6931 | Sodium valproate |
| 0.6930 | Atenolol |
| 0.6926 | Cimetidine |
| 0.6926 | Pyrilamine |
| 0.6922 | Desmethylclobazam |
| 0.6922 | N-Desmethylclobazam |
| 0.6913 | Codeineb |
| 0.6913 | Hexobarbital |
| 0.6901 | a-Hydroxyalprazolam |
| 0.6893 | Tiotidinec |
| 0.6860 | Ibuprofen |
| 0.6583 | Antipyrine |
| 0.6580 | Ether |
| 0.6508 | Nor-1-Chlorpromazine |
| 0.6499 | Mesoridazine |
| 0.6060 | Trichloroethene |
| 0.5607 | 1,1,1-Trichloroethane |
| 0.5467 | Hydroxyzine |
| 0.5183 | Carbamazepine-EPO |
| 0.5036 | 2-Chloro-1,1,1-Trifluoroethane |
| 0.4505 | p-Xylene |
| 0.4433 | Chloroform |
| 0.4382 | Toluene |
| 0.4345 | Dichloromethane |
| 0.3648 | Methane |
| 0.3604 | Theophyline |
| 0.3064 | 2-Propanol |
| 0.2989 | Cyclohexane |
| 0.2471 | m-Xylene |
| 0.2456 | Sulfur hexafluoride |
| 0.2265 | Paracetamol |
| 0.2159 | Divinyl eter |
| 0.2118 | Pentane |
| 0.1958 | Ethanol |
| 0.1904 | Actylsalicylic acid |
| 0.1853 | Methoxyflurane |
| 0.1788 | 3-Methylhexane |
| 0.1755 | Caffeine |
| 0.1754 | Salicylic acid |
| 0.1331 | R-Enflurane |
| 0.0527 | Risperidone |
| 0.0448 | Butanone |
| 0.0221 | Zidovudine |
| 0.0117 | Fluphenazinec |
| 0.0064 | Domperidoneb |
| 0.0062 | Nevirapine |
| 0.0061 | Amitriptylineb |
| 0.0045 | Physostigmine |
| 0.0044 | Morphineb |
| 0.0043 | Phencyclidine |
| 0.0042 | Indomethacinc |
| 0.0040 | Pentobarbital |
| 0.0038 | 1-Hydroxymidazolam |
| 0.0033 | Heptane |
| 0.0031 | Albuterolb |
| 0.0026 | Alprazolam |
| 0.0002 | Zolantidineb |
| 0.0002 | Buspirone |
| 0.0002 | Icotidineb,c |

**Table S1.** BBB dataset similarity.


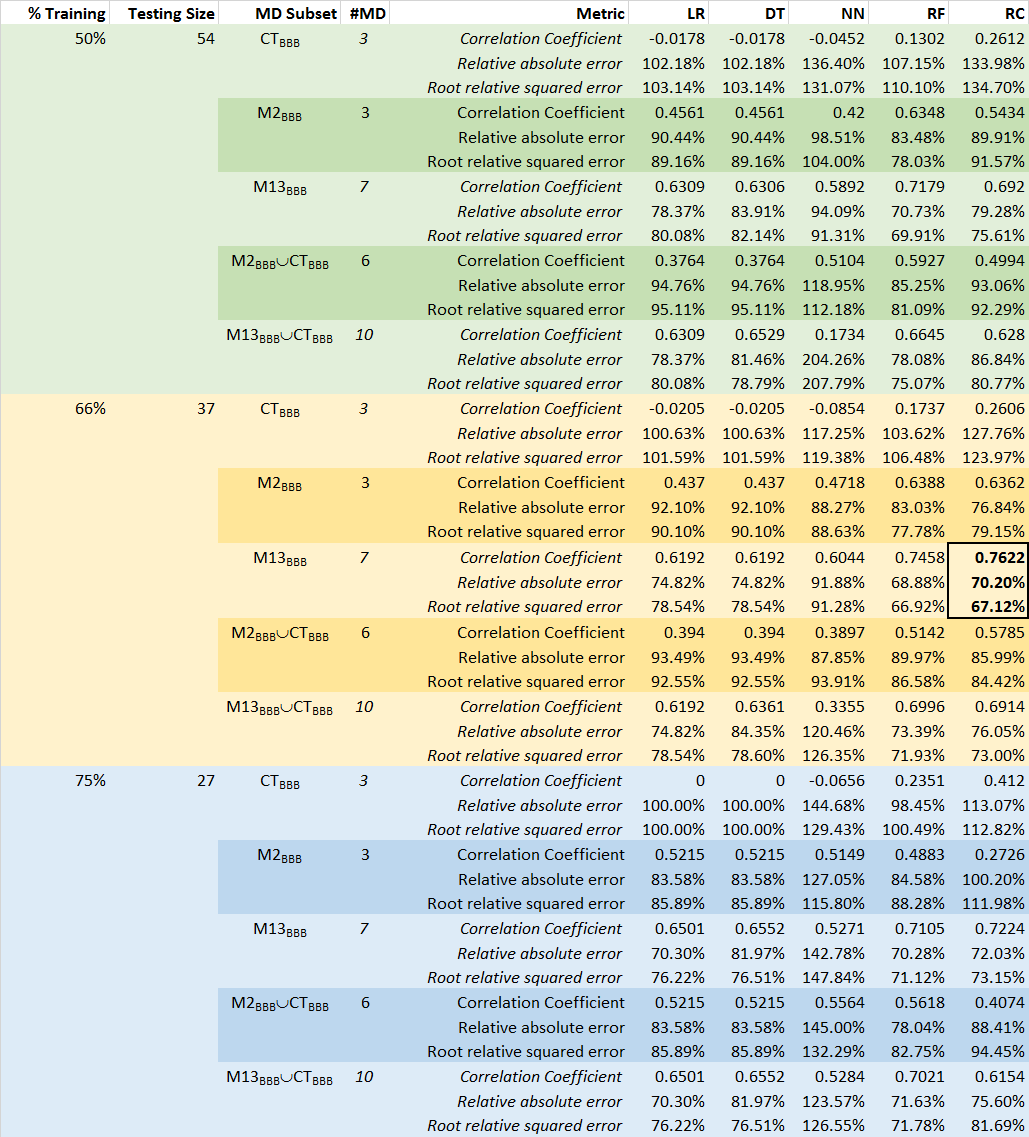


**Table S2. Part A.** Performances of Regression QSAR Models for BBB dataset. The result highlighted in bold corresponds to the most accurate regression model.


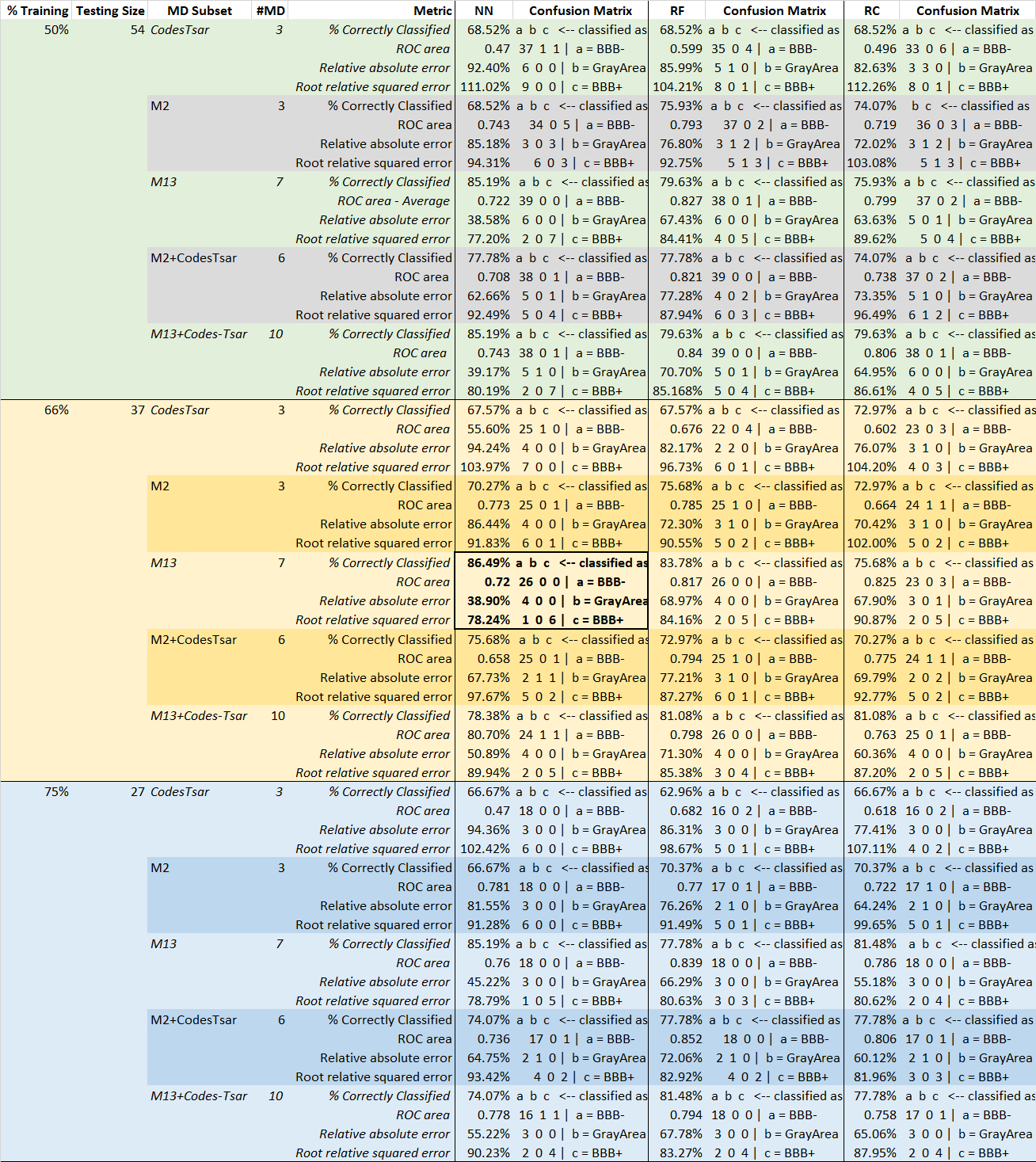


**Table S2. Part B.** Performances of Classification QSAR Models for BBB dataset. The result highlighted in bold corresponds to the most accurate classification model. Classes: High (BBB-), Med (Gray Area), Low (BBB+).

| 1.0000 | Acrivastine |
| --- | --- |
| 1.0000 | Granisetron |
| 1.0000 | Lisinopril |
| 0.9993 | Stavudine |
| 0.9991 | Captopril |
| 0.9990 | Cefuroxime |
| 0.9989 | Venlafaxine |
| 0.9984 | AAFC |
| 0.9980 | Reproterol |
| 0.9977 | Nisoldipine |
| 0.9973 | Doxorubicin |
| 0.9972 | Lamivudine |
| 0.9963 | Moxonidine |
| 0.9953 | Atropine |
| 0.9948 | Eflornithine |
| 0.9947 | Mexiletine |
| 0.9943 | Alprazolam |
| 0.9937 | Bretylium |
| 0.9930 | Viloxazine |
| 0.9929 | Oxprenolol |
| 0.9929 | Phenoxymethyl |
| 0.9926 | Naloxone |
| 0.9925 | Naproxen |
| 0.9924 | D-Phe-1-Pro |
| 0.9923 | Pindolol |
| 0.9923 | Metoprolol |
| 0.9923 | Ketorolac |
| 0.9922 | Loracarbef |
| 0.9922 | Chloramphenicol |
| 0.9922 | Ethambutol |
| 0.9921 | Cefuroxime |
| 0.9921 | Ceftizoxime |
| 0.9921 | Timolol |
| 0.9920 | Indomethacin |
| 0.9920 | Piroxicam |
| 0.9919 | Metolazone |
| 0.9919 | Nitrendipine |
| 0.9918 | Pefloxacin |
| 0.9918 | Morphine |
| 0.9917 | Propanolol |
| 0.9914 | Pirbuterol |
| 0.9912 | Tramadol |
| 0.9912 | Ziprasidone |
| 0.9912 | Tranexamic |
| 0.9909 | Gabapentin |
| 0.9909 | Imipramine |
| 0.9907 | Trapidil |
| 0.9907 | Tenidap |
| 0.9907 | Ximoprofen |
| 0.9900 | Metaproterenol |
| 0.9898 | Torasemide |
| 0.9896 | Mifobate |
| 0.9894 | Minoxidil |
| 0.9893 | Neomycin |
| 0.9893 | Tiagabine |
| 0.9870 | Hydrochlorothiazide |
| 0.9864 | Testosterone |
| 0.9856 | Guanoxan |
| 0.9847 | Disulfiram |
| 0.9728 | Betaxolol |
| 0.9701 | Zidovudine |
| 0.9632 | Dihydrocodeine |
| 0.9360 | Pafenolol |
| 0.9234 | Cymarin |
| 0.9150 | Vigabatrin |
| 0.8431 | Caffeine |
| 0.8290 | Acetylsalicylic |
| 0.8288 | Amrinone |
| 0.8125 | Lamotrigine |
| 0.8058 | Salicylic |
| 0.7944 | Paracetamol |
| 0.7694 | Saccharin |
| 0.7681 | Ascorbic |
| 0.7525 | Chlorothiazide |
| 0.7260 | Sorivudine |
| 0.7156 | Isoniazid |
| 0.6663 | D-Amphetamine |
| 0.6614 | Amiloride |
| 0.6497 | Foscarnet |
| 0.6449 | Metformin |
| 0.6158 | Theophyline |
| 0.5918 | Oxatomide |
| 0.5831 | Cycloserine |
| 0.5755 | Guanabenz |
| 0.5607 | Lincomycin |
| 0.5363 | Distigmine |
| 0.4899 | Nefazodone |
| 0.4698 | Chloroform |
| 0.4525 | Nicotinic |
| 0.3854 | Recainam |
| 0.3742 | Acebutolol |
| 0.3654 | Anipyrine |
| 0.3536 | Iothalamate |
| 0.3497 | Clofibrate |
| 0.3466 | Adefovir |
| 0.3463 | Nizatidine |
| 0.3413 | Dexamethasone |
| 0.3401 | Ribavirin |
| 0.3385 | Flecainide |
| 0.3382 | Methadone |
| 0.3364 | Methyldopa |
| 0.3340 | Rimiterol |
| 0.3323 | Meloxicam |
| 0.3318 | Ketoprofen |
| 0.3316 | Praziquantel |
| 0.3316 | Sudoxicam |
| 0.3315 | Isoxicam |
| 0.3313 | Practolol |
| 0.3311 | Famciclovir |
| 0.3306 | Labetalol |
| 0.3306 | Ciprofloxacin |
| 0.3306 | Cimetidine |
| 0.3302 | Norfloxacin |
| 0.3302 | Sodium |
| 0.3302 | Atenolol |
| 0.3295 | Terazocin |
| 0.3293 | Oxazepam |
| 0.3292 | Felbamate |
| 0.3288 | Trimethoprim |
| 0.3288 | Ondansetron |
| 0.3286 | Phenglutarimide |
| 0.3283 | Gliclazide |
| 0.3279 | Levodopa |
| 0.3277 | Fluconazole |
| 0.3263 | Fenoterol |
| 0.3260 | Famotidine |
| 0.3246 | Sotalol |
| 0.3245 | Aztreonam |
| 0.3243 | Terbutaline |
| 0.3225 | Diazepam |
| 0.3219 | Aciclovir |
| 0.3127 | Furosemide |
| 0.3088 | Propiverine |
| 0.3069 | Progesterone |
| 0.3012 | Trovafloxacin |
| 0.2935 | Prednisolone |
| 0.2876 | Enalaprilat |
| 0.2821 | Sulpiride |
| 0.2776 | Urapidil |
| 0.2743 | Methylprednisolone |
| 0.2562 | Sultopride |
| 0.2542 | Glyburide |
| 0.2443 | Ouabain |
| 0.2281 | Prazosin |
| 0.2232 | Methotrexate |
| 0.2162 | Pravastatin |
| 0.2092 | Piroximone |
| 0.2043 | Propylthiouracil |
| 0.1968 | Cefatrizine |
| 0.1915 | Carfecillin |
| 0.1877 | Lactulose |
| 0.1850 | Olsalazine |
| 0.1843 | Nordazepam |
| 0.1836 | Bromazepam |
| 0.1835 | Cromolym |
| 0.1830 | Felodipine |
| 0.1829 | Scopolamine |
| 0.1826 | Etoposide |
| 0.1812 | Tolmesoxide |
| 0.1803 | Sumatriptan |
| 0.1797 | Netivudine |
| 0.1793 | Desipramine |
| 0.1789 | Ethynylestradiol |
| 0.1787 | Tolbutamide |
| 0.1783 | Lormetazepan |
| 0.1780 | Cefadroxil |
| 0.1768 | Phenytoin |
| 0.1767 | Topiramate |
| 0.1766 | Ampicillin |
| 0.1762 | Alprenolol |
| 0.1762 | Cephalexin |
| 0.1761 | Amoxicillin |
| 0.1760 | Lornoxicam |
| 0.1754 | Sulindac |
| 0.1747 | Bumetanide |
| 0.1743 | Azosemide |
| 0.1743 | Quinidine |
| 0.1721 | Ofloxacin |
| 0.1712 | Mannitol |
| 0.1706 | Tenoxicam |
| 0.1706 | Nadolol |
| 0.1692 | Sulphasalazine |
| 0.1689 | Benzylpenicillin |
| 0.1670 | Levonorgestrel |
| 0.1623 | Ceftriaxone |
| 0.1610 | Enalapril |
| 0.1588 | Spironolactone |
| 0.1328 | Camazepam |
| 0.1296 | Lansoprazole |
| 0.1206 | Toremifene |
| 0.0984 | Oxyfedrine |
| 0.0878 | Clonidine |
| 0.0789 | Flumenethyl |
| 0.0503 | Gallopamil |
| 0.0457 | Telmisartan |
| 0.0456 | Cisapride |
| 0.0434 | Fosfomycin |
| 0.0391 | Verapamil |
| 0.0230 | Fosmidomycin |
| 0.0160 | Mibefradil |
| 0.0090 | Mercaptoethanesulfonic |
| 0.0021 | Glycine |

**Table S3.** HIA dataset similarity.


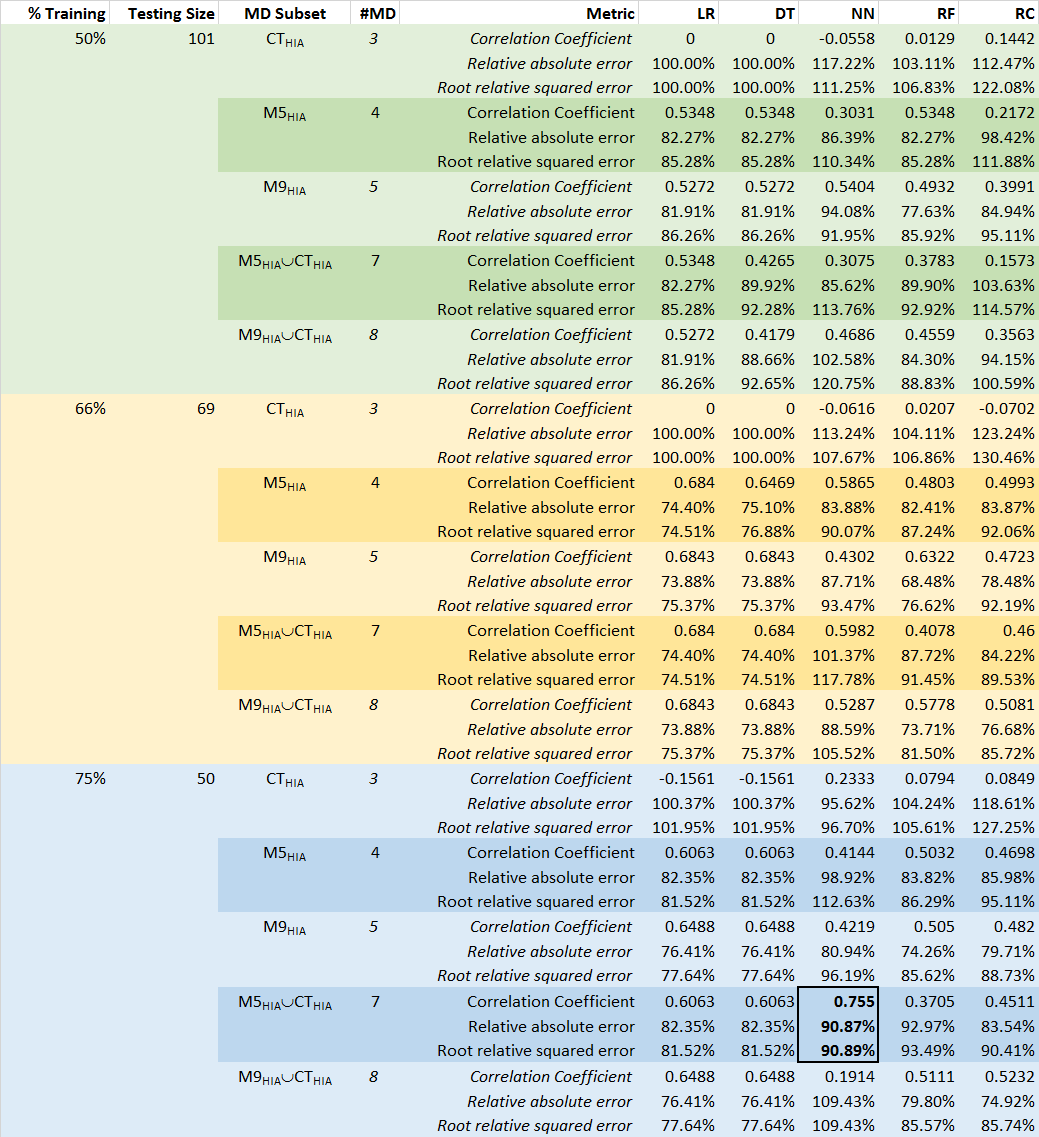


**Table S4. Part A.** Performances of Regression QSAR Models for HIA dataset. The result highlighted in bold corresponds to the most accurate regression model.


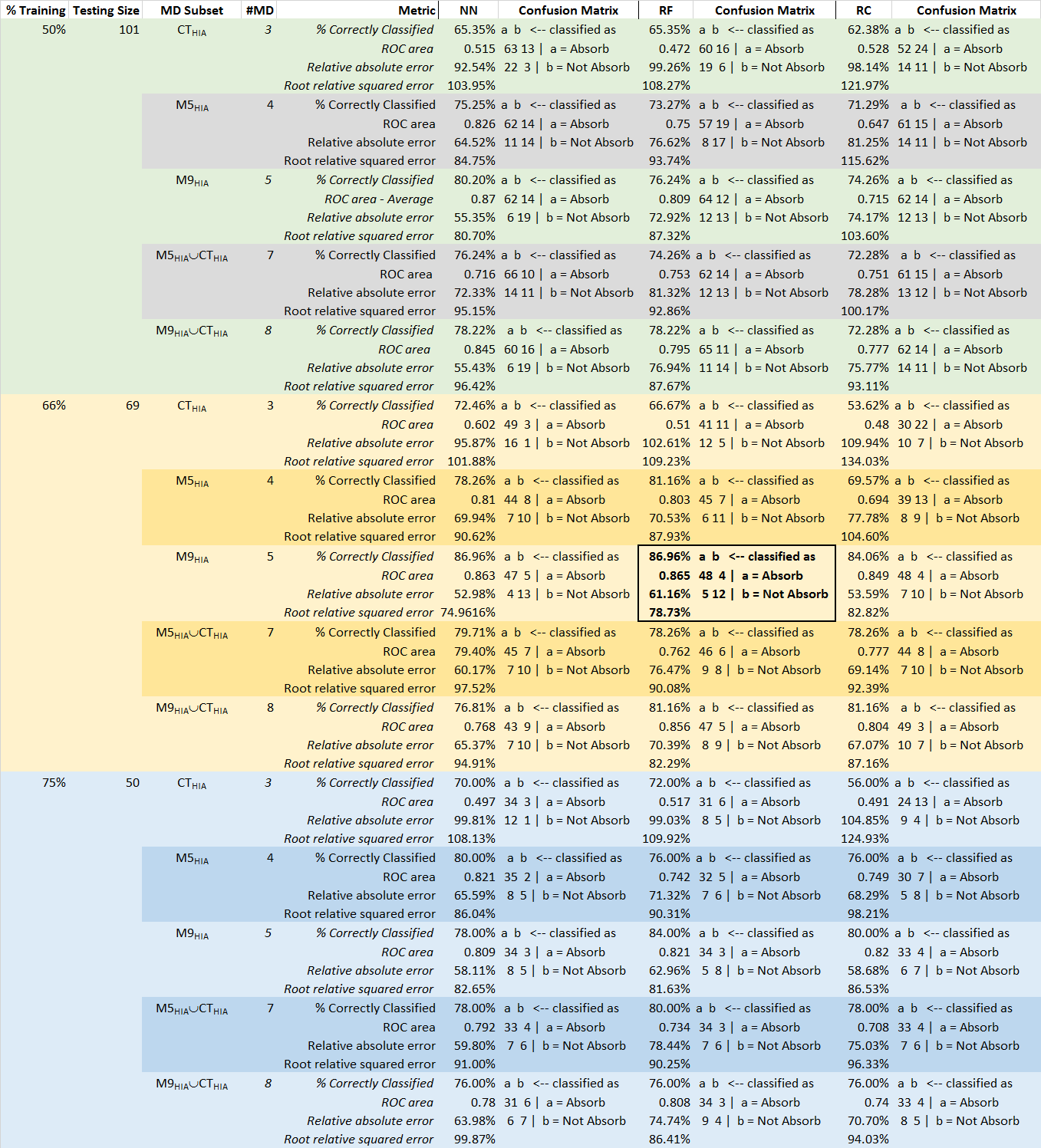


**Table S4. Part B.** Performances of Classification QSAR Models for HIA dataset. The result highlighted in bold corresponds to the most accurate classification model.

| 1 | 0.10479 |
| --- | --- |
| 2 | 0.12695 |
| 3 | 0.24374 |
| 4 | 0.24476 |
| 5 | 0.25267 |
| 6 | 0.26369 |
| 7 | 0.26376 |
| 8 | 0.26871 |
| 9 | 0.26915 |
| 10 | 0.26938 |
| 11 | 0.27519 |
| 12 | 0.27806 |
| 13 | 0.27905 |
| 14 | 0.29055 |
| 15 | 0.29479 |
| 16 | 0.29858 |
| 17 | 0.29964 |
| 18 | 0.30257 |
| 19 | 0.30305 |
| 20 | 0.30595 |
| 21 | 0.30924 |
| 22 | 0.31468 |
| 23 | 0.32190 |
| 24 | 0.32522 |
| 25 | 0.32573 |
| 26 | 0.32587 |
| 27 | 0.32667 |
| 28 | 0.32774 |
| 29 | 0.32788 |
| 30 | 0.32807 |
| 31 | 0.33157 |
| 32 | 0.33253 |
| 33 | 0.33507 |
| 34 | 0.33525 |
| 35 | 0.33533 |
| 36 | 0.33646 |
| 37 | 0.33845 |
| 38 | 0.33863 |
| 39 | 0.33986 |
| 40 | 0.34026 |
| 41 | 0.34145 |
| 42 | 0.34281 |
| 43 | 0.34339 |
| 44 | 0.34449 |
| 45 | 0.34454 |
| 46 | 0.34483 |
| 47 | 0.34509 |
| 48 | 0.34603 |
| 49 | 0.34612 |
| 50 | 0.34663 |
| 51 | 0.34733 |
| 52 | 0.34740 |
| 53 | 0.34745 |
| 54 | 0.34801 |
| 55 | 0.34825 |
| 56 | 0.34881 |
| 57 | 0.34883 |
| 58 | 0.34885 |
| 59 | 0.34885 |
| 60 | 0.34914 |
| 61 | 0.35029 |
| 62 | 0.35074 |
| 63 | 0.35166 |
| 64 | 0.35343 |
| 65 | 0.35380 |
| 66 | 0.35409 |
| 67 | 0.35423 |
| 68 | 0.35429 |
| 69 | 0.35475 |
| 70 | 0.35529 |
| 71 | 0.35673 |
| 72 | 0.35756 |
| 73 | 0.35836 |
| 74 | 0.36046 |
| 75 | 0.36147 |
| 76 | 0.36259 |
| 77 | 0.36324 |
| 78 | 0.36526 |
| 79 | 0.36598 |
| 80 | 0.36810 |
| 81 | 0.36893 |
| 82 | 0.36894 |
| 83 | 0.37263 |
| 84 | 0.37490 |
| 85 | 0.37534 |
| 86 | 0.38506 |
| 87 | 0.39019 |
| 88 | 0.39771 |
| 89 | 0.39779 |
| 90 | 0.39779 |
| 91 | 0.39779 |
| 92 | 0.40284 |
| 93 | 0.40433 |
| 94 | 0.41114 |
| 95 | 0.41269 |
| 96 | 0.42745 |
| 97 | 0.42761 |
| 98 | 0.43096 |
| 99 | 0.43097 |
| 100 | 0.43113 |
| 101 | 0.43284 |
| 102 | 0.43420 |
| 103 | 0.43507 |
| 104 | 0.43531 |
| 105 | 0.43973 |
| 106 | 0.44139 |
| 107 | 0.44240 |
| 108 | 0.44358 |
| 109 | 0.44540 |
| 110 | 0.44550 |
| 111 | 0.44675 |
| 112 | 0.46180 |
| 113 | 0.46297 |
| 114 | 0.46781 |
| 115 | 0.47191 |
| 116 | 0.48427 |
| 117 | 0.48476 |
| 118 | 0.48959 |
| 119 | 0.49213 |
| 120 | 0.49422 |
| 121 | 0.49516 |
| 122 | 0.49588 |
| 123 | 0.49893 |
| 124 | 0.50176 |
| 125 | 0.50899 |
| 126 | 0.51093 |
| 127 | 0.51649 |
| 128 | 0.55787 |
| 129 | 0.55836 |
| 130 | 0.57937 |
| 131 | 0.58994 |
| 132 | 0.59746 |
| 133 | 0.62998 |
| 134 | 0.63255 |
| 135 | 0.66637 |
| 136 | 0.67479 |
| 137 | 0.68982 |
| 138 | 0.69075 |
| 139 | 0.71198 |
| 140 | 0.71680 |
| 141 | 0.72478 |
| 142 | 0.73075 |
| 143 | 0.73324 |
| 144 | 0.73928 |
| 145 | 0.74534 |
| 146 | 0.75007 |
| 147 | 0.75853 |
| 148 | 0.76104 |
| 149 | 0.76562 |
| 150 | 0.77541 |
| 151 | 0.77704 |
| 152 | 0.79219 |
| 153 | 0.80883 |
| 154 | 0.81209 |
| 155 | 0.83162 |
| 156 | 0.83184 |
| 157 | 0.83619 |
| 158 | 0.83779 |
| 159 | 0.84705 |
| 160 | 0.87088 |
| 161 | 0.87514 |
| 162 | 0.87805 |
| 163 | 0.88339 |
| 164 | 0.90187 |
| 165 | 0.92367 |
| 166 | 0.93324 |
| 167 | 0.95861 |
| 168 | 0.96045 |
| 169 | 0.96875 |
| 170 | 0.98151 |
| 171 | 0.98203 |
| 172 | 0.98362 |
| 173 | 0.98533 |
| 174 | 0.98760 |
| 175 | 0.99007 |
| 176 | 0.99059 |
| 177 | 1.00000 |

**Table S5.** EE dataset similarity.


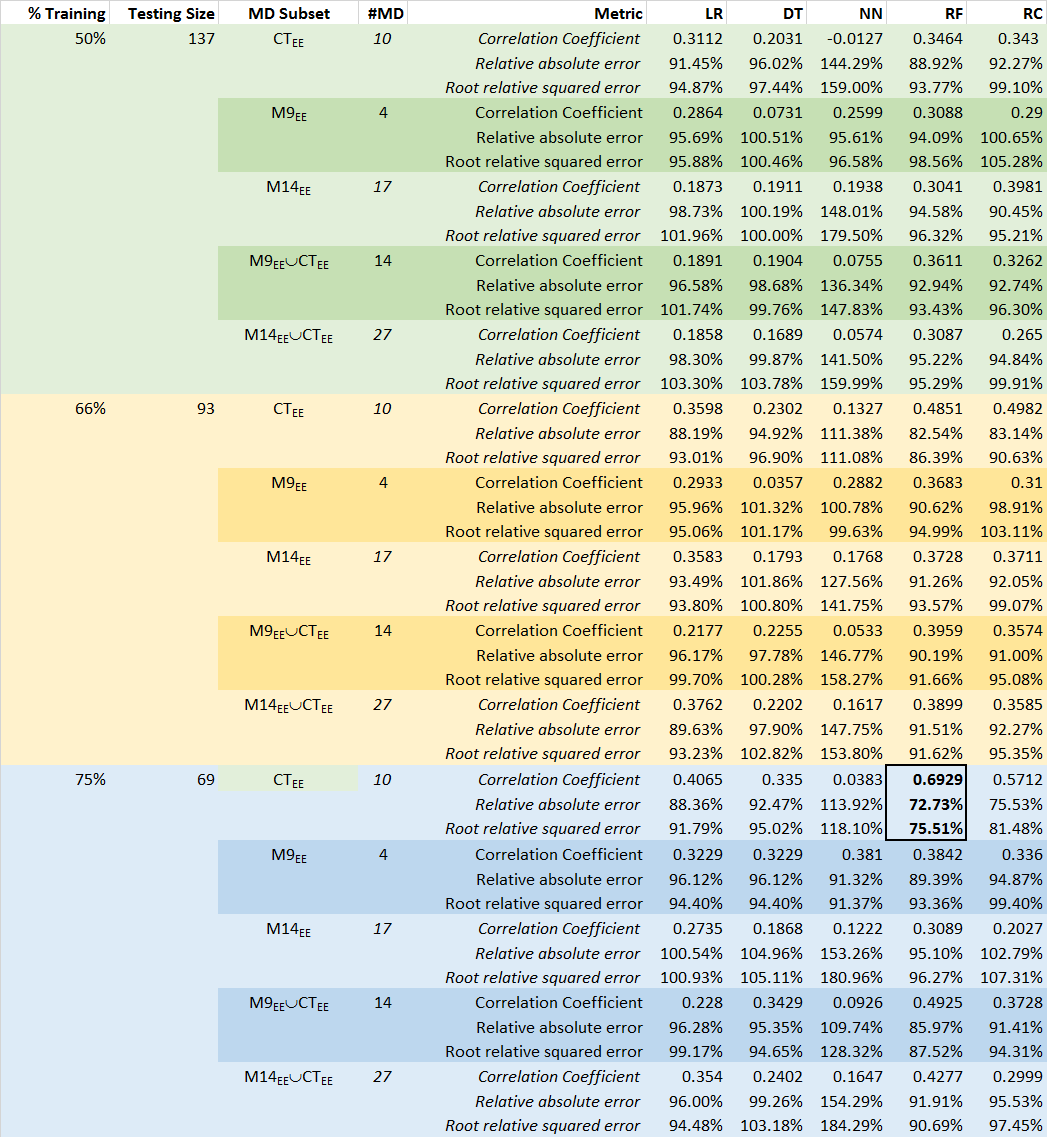


**Table S6. Part A.** Performances of Regression QSAR Models for EE dataset. The result highlighted in bold corresponds to the most accurate regression model.


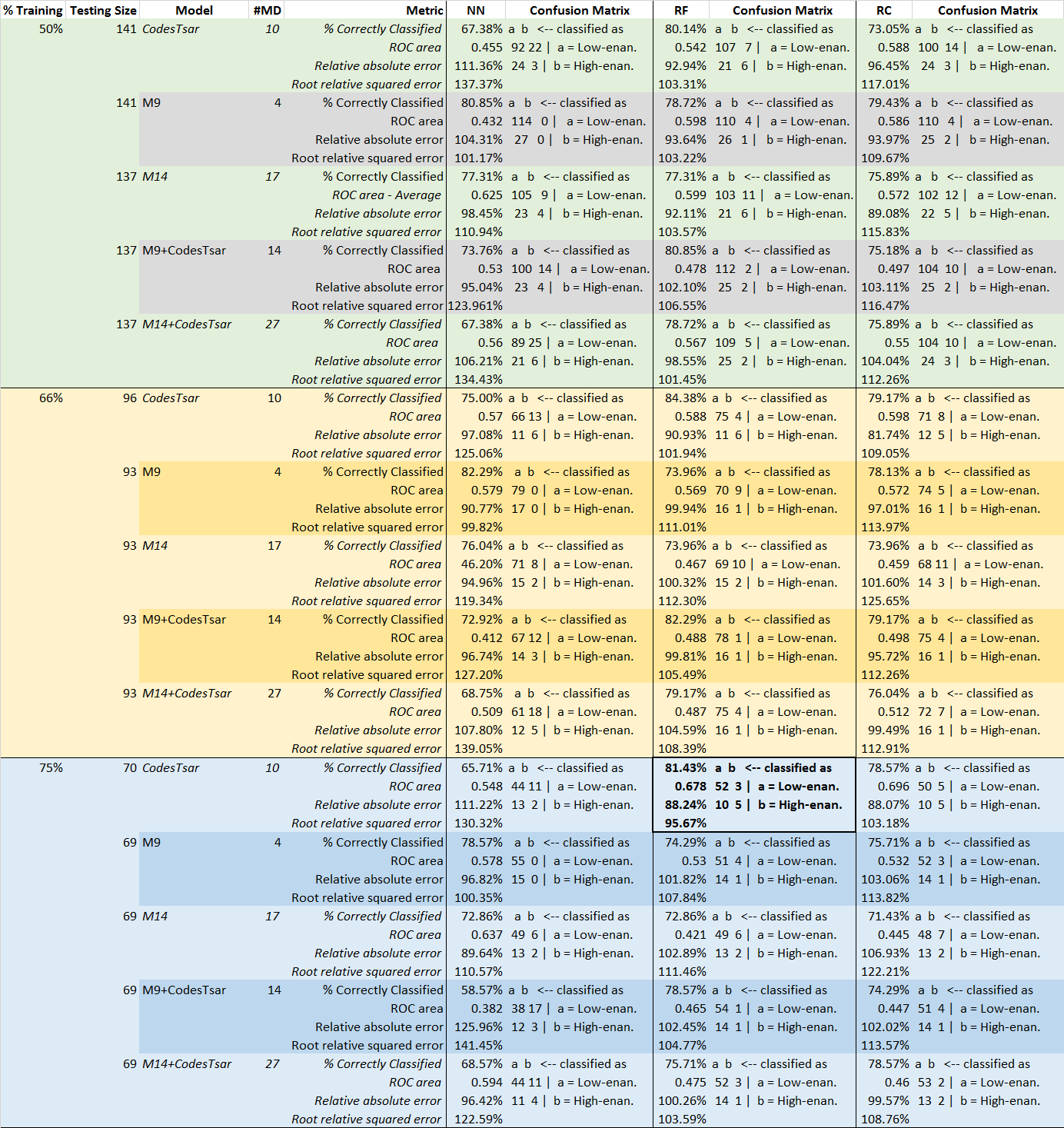


**Table S6. Part B.** Performances of Classification QSAR Models for EE dataset. The result highlighted in bold corresponds to the most accurate classification model.
